# A Two-Staged Model of Na^+^ Exclusion in Rice Explained by 3D Modeling of HKT Transporters and Alternative Splicing

**DOI:** 10.1371/journal.pone.0039865

**Published:** 2012-07-11

**Authors:** Olivier Cotsaftis, Darren Plett, Neil Shirley, Mark Tester, Maria Hrmova

**Affiliations:** Australian Centre for Plant Functional Genomics, University of Adelaide, Adelaide, South Australia, Australia; United States Department of Agriculture, Agricultural Research Service, United States of America

## Abstract

The HKT family of Na^+^ and Na^+^/K^+^ transporters is implicated in plant salinity tolerance. Amongst these transporters, the cereal HKT1;4 and HKT1;5 are responsible for Na^+^ exclusion from photosynthetic tissues, a key mechanism for plant salinity tolerance. It has been suggested that Na^+^ is retrieved from the xylem transpiration stream either in the root or the leaf sheath, protecting the leaf blades from excessive Na^+^ accumulation. However, direct evidence for this scenario is scarce. Comparative modeling and evaluation of rice (Oryza sativa) HKT-transporters based on the recent crystal structure of the bacterial TrkH K^+^ transporter allowed to reconcile transcriptomic and physiological data. For OsHKT1;5, both transcript abundance and protein structural features within the selectivity filter could control shoot Na^+^ accumulation in a range of rice varieties. For OsHKT1;4, alternative splicing of transcript and the anatomical complexity of the sheath needed to be taken into account. Thus, Na^+^ accumulation in a specific leaf blade seems to be regulated by abundance of a correctly spliced OsHKT1;4 transcript in a corresponding sheath. Overall, allelic variation of leaf blade Na^+^ accumulation can be explained by a complex interplay of gene transcription, alternative splicing and protein structure.

## Introduction

Exclusion of Na^+^ from shoot tissue is an important mechanism required by plants to tolerate saline growth conditions [1]. The HKT family of transporters is considered crucial to the process of maintaining low shoot tissue Na^+^ concentration [2], [3]. The HKT1;4 and HKT1;5 transporters were suggested to be particularly important in the retrieval of Na^+^ from the transpiration stream, a process which prevents further transport of Na^+^ to the leaves [4]. These transporters have been particularly well characterized in durum wheat and have been implicated as the candidate genes for the Na^+^-exclusion loci Nax1 and Nax2 [5]. The transporter encoded by the candidate gene for Nax1, HKT1;4, has been shown to be responsible for retrieval of Na^+^ from the transpiration stream for storage in the leaf sheath tissue [6]. The transporter encoded by the Nax2 candidate, HKT1;5, plays a similar role, but appears to function primarily in root tissue [7]. Targeted overexpression of the Arabidopsis HKT1;5 homolog, AtHKT1;1, in Arabidopsis and rice has been shown to increase Na^+^ exclusion from the shoot [8], [9]. Recent work describes the inclusion of an ancestral HKT1;5 gene (Nax2) into modern wheat varieties and highlights the significant salinity tolerance and yield improvement that results in a saline field environment [10], [11].

The diversity of salinity tolerance amongst rice lines is a well described phenomenon [12], [13]. A mapping population developed between the salinity tolerant variety Nona Bokra and a salinity sensitive variety Koshihikari identified the SKC1 quantitative trait locus (QTL) as critical for Na^+^ exclusion [14]. This work identified OsHKT1;5 as the candidate gene for this QTL and described the allelic differences potentially responsible for the increased Na^+^ transport efficiency of the Nona Bokra allele.

The work described here provides evidence for a crucial amino acid substitution which determines Na^+^ transport efficiency of OsHKT1;5. The recently solved structure of a highly similar TrkH K^+^ transporter [15] provided the basis for three-dimensional (3D) molecular modeling of OsHKT1;5 and OsHKT1;4. Additionally, evidence is provided for the involvement of an alternative splicing mechanism for correct function of OsHKT1;4 in Na^+^ retrieval into leaf sheath tissue. Finally, gene transcription, alternative splicing and protein structure data are incorporated into a theoretical model which describes the process of Na^+^ exclusion in rice.

## Results and Discussion

### Root-to-shoot Na^+^ transfer in rice upon salt stress

**Figure 1:**
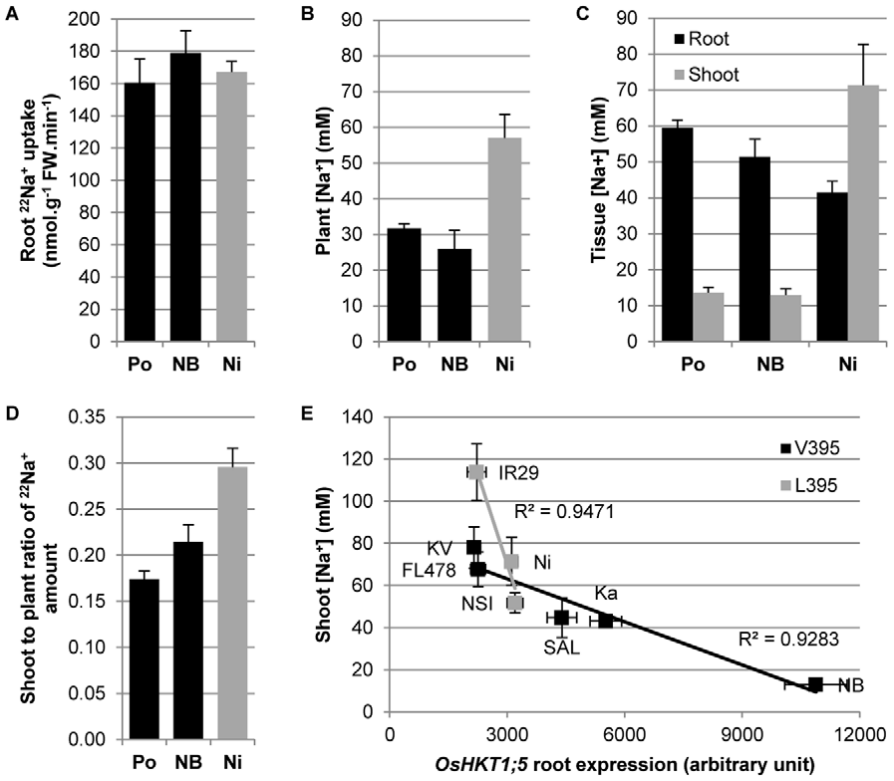
Root-to-shoot Na^+^ transfer control in rice upon salt stress. (**A**) Radioactive ^22^Na^+^ fluxes measurement of root Na^+^ uptake (+SEM; n = 8) (uptake: 2 min). (**B**) Flame photometry measurement of plant total Na^+^ concentration (+SEM; n = 5). (**C**) Flame photometry measurement of tissue Na^+^ concentrations (+SEM; n = 5). (**D**) Radioactive ^22^Na^+^ fluxes measurement of the shoot-to-plant ratio of Na^+^ amount (+SEM; n = 8). (**E**) Correlation between root OsHKT1;5 expression level measured by qRT-PCR (three biological replicates per line +/− SEM; three technical replicates per biological sample) and shoot Na^+^ concentration measured by flame photometry (+/− SEM; n = 5) in salt treated rice lines with either a Leu or a Val residue in position 395 of the OsHKT1;5 gene. The abbreviations Po = Pokkali; NB = Nona Bokra; Ni = Nipponbare; KV = Kallurundai Vellai; Ka = Kalurundai; NSI = NSICRC106; and SAL = SAL208 stand for individual varieties, variously represented through the panels A–E. IR29 and FL478 are full names of rice varieties.

**Figure 1a, b, c, d** illustrates the behavior under salt stress of Pokkali and Nona Bokra, two classical salt-tolerant indica landraces, and Nipponbare, a widely used salt-sensitive japonica cultivar. Despite the larger mass of the tolerant landraces, and similar Na^+^ uptake rates from the external growth medium across all three lines (**Figure 1a**), both landraces are able to maintain a lower total plant Na^+^ concentration than Nipponbare (**Figure 1b**). Moreover, although the landraces sequester more Na^+^ in the root, and potentially release part of it back to the external medium, Nipponbare allows for more Na^+^ transfer to the shoot via the transpiration stream (**Figure 1c**). Radioactive ^22^Na^+^ flux experiments confirmed this observation, with Nipponbare having a higher root-to-shoot transfer of ^22^Na^+^ than Pokkali or Nona Bokra (**Figure 1d**).

### Molecular modeling of the OsHKT1;5 transporter

**Figure 2:**
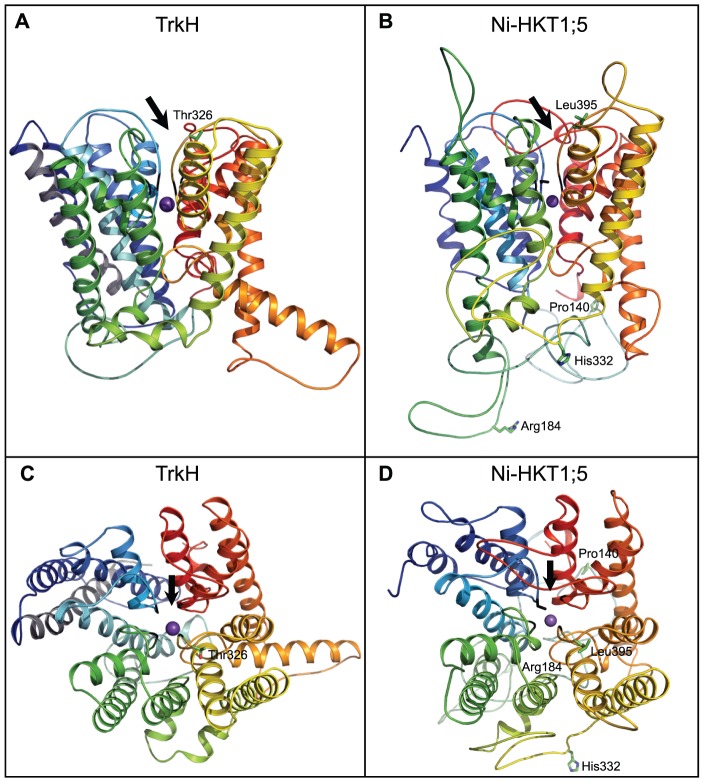
Crystal structure of a TrkH (3PJZ) and molecular model of the rice OsHKT1;5 transporter. (**A–B**) Cartoon representations of a bacterial TrkH K^+^ transporter and a model of Ni-OsHKT1;5 illustrating the overall folds of transporters (Ni = Nipponbare). Both structures are coloured in rainbow, except helix 0 in TrkH that is in grey and that is absent in Ni-OsHKT1;5. Four variations between Ni-OsHKT1;5 and Po-OsHKT1;5 (Po = Pokkali) are indicated in green cpk (A140P, H184R, D332H and V395L). Thr328 in TrkH (A) corresponds to Leu395 in Ni-OsKHT1;5 (B). Black lines indicate the Gly tetrad in TrkH, while the Ser-Gly-Gly-Gly signature is present in Ni-OsHKT1;5. Purple spheres indicate K^+^ (TrkH) and Na^+^ (Ni-OsHKT1;5) ions. The entry into the pores of transporters is indicated by black arrows. (**C–D**) The view in A–B is rotated by 90 degrees along the x-axis and shows TrkH and Ni-HKT1;5 viewed from a cytoplasmic side.

It was demonstrated that OsHKT1;5 is an important transporter controlling shoot Na^+^ concentration in rice [14]. Furthermore, two recent studies showed that the targeted over-expression of AtHKT1;1, the Arabidopsis HKT1;5-homolog, in Arabidopsis or rice roots resulted in a higher Na^+^ exclusion from the shoot [8], [9]. To better understand the function of HKT1;5-like transporters, we constructed a 3D molecular model of the Nipponbare (Ni) OsHKT1;5 transporter (GenBank accession BAF04762; **Figure 2**), based on its structural similarity with a recently solved TrkH K^+^ transporter [15] (**Figure S1**). This type of structural analysis and comparison were not possible before as the 3D structure of the TrkH K^+^ transporter was only recently solved [15]. While it is expected that the helical bundle component of the rice Ni-OsHKT1;5 transporter has been reliably predicted, the positions of loops inter-connecting individual α-helices might have lower structural reliability, in particular when these loops are longer than approximately twelve residues. The Ni-OsHKT1;5 model was then extended to the allelic version of HKT1;5 found in Pokkali (Po-OsHKT1;5; GenBank accession ABN48306), whose amino acid sequence differs from Ni-OsHKT1;5 in four residues: A140P, H184R, D332H and V395L (**Figures 2** and **S1**). These variations are identical to the ones previously identified when the sequences of the Nipponbare-like Koshihikari and Pokkali-like Nona Bokra OsHKT1;5's were compared [14].

**Figure 3:**
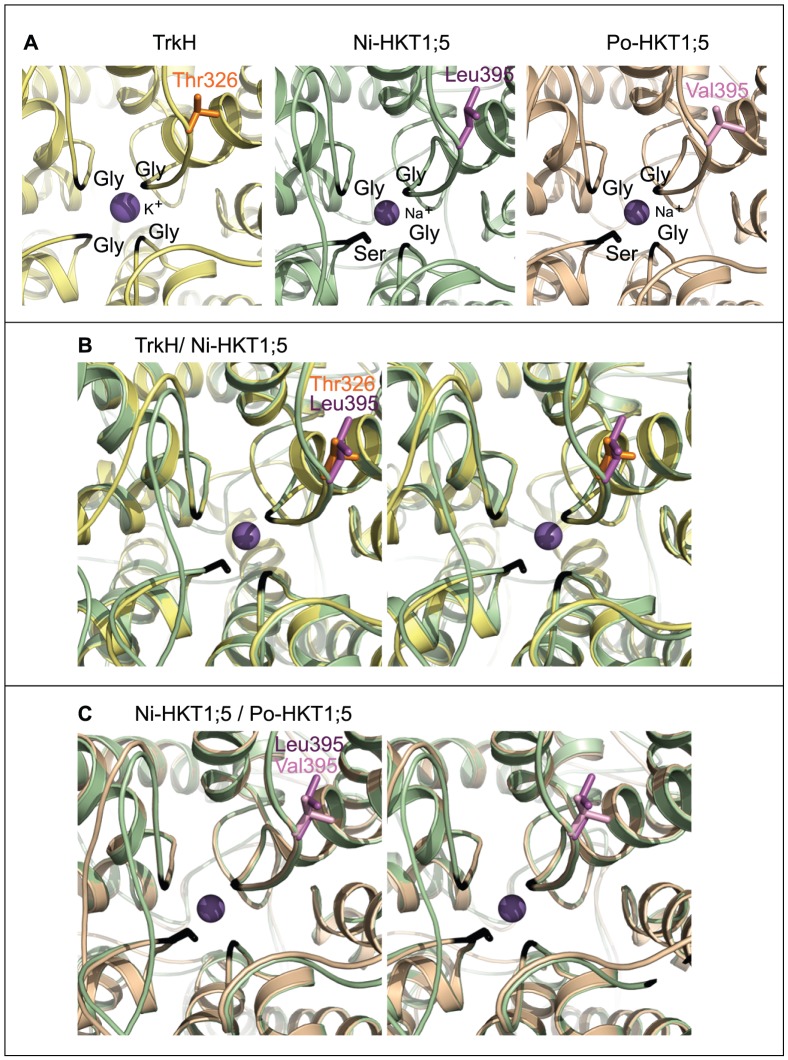
Selectivity filters of TrkH and the rice Ni-OsHKT1;5 and Po-OsHKT1;5 transporters. (**A**) Views of the pore regions with K^+^ (TrkH) and Na^+^ (Ni-OsHKT1;5 and Po-OsHKT1;5) ions coloured in purple spheres (Ni = Nipponbare; and Po = Pokkali). The Gly-Gly-Gly-Gly (TrkH) and Ser-Gly-Gly-Gly (Ni-OsHKT1;5 and Po-OsHKT1;5) signatures are coloured in black. The residue (stick) at the entrance of the pore in TrkH is Thr326 (orange), while Leu395 (dark magenta) is present in Ni-OsHKT1;5, and Val395 (light magenta) occurs in Po-OsHKT1;5. These residues are likely to affect pore rigidity and dispositions of residues controlling the rates of Na^+^ transport. (**B**) A stereo superposition of TrkH (yellow) and Ni-OsHKT1;5 (green). (**C**) A stereo superposition of Ni-OsHKT1;5 (green) and Po-OsHKT1;5 (wheat).

The 3D models of Ni-OsHKT1;5 and Po-OsHKT1;5 show the presence of three glycine residues (Gly264, Gly391 and Gly495) and one serine residue (Ser76) forming a selectivity filter (**Figure 3**) that delineates the entry into the pore of both transporters (**Figure 2**; black arrows). This Ser-Gly-Gly-Gly signature suggests that both Ni-OsHKT1;5 and Po-OsHKT1;5 have a potential to mediate the selective transport of Na^+^ over K^+^, due to a steric hindrance imposed through Ser76 that might cause for K^+^ (with a larger atomic radius than Na^+^) to be transported unfavorably.

Further, the 3D models of Ni-OsHKT1;5 (associated with slower transport rates; **Figure 1d**) and Po-OsHKT1;5 (associated with faster transport rates; **Figure 1d**) indicate that the first three substitutions between the two transporters – A140P, H184R and D332H – are positioned on the cytoplasm-exposed loops inter-connecting membrane α-helices, whereas the V395L substitution is positioned in the vicinity of the selectivity filter (**Figures 2** and **3**). We surmised that it is unlikely that the first three substitutions would significantly interfere with Na^+^ transport in both rice transporters. However, the V395L substitution is positioned strategically in the close proximity of Gly391 near the entrance of the pore in both transporters (respective dark and light purple coloring of Leu395 and Val395 in **Figure 3**). Thus, we propose that the V395L point mutation in Ni-OsHKT1;5 could directly affect pore rigidity, due to a larger van der Waals volume imposed by the side-chain of Leu395 compared to that of Val395, and thus slow down Na^+^ transport rates. The V395L substitution in Ni-OsHKT1;5 could also influence the dispositions of other key residues underlying the ion selectivity within the pore environment. Nevertheless, the ultimate confirmation of these hypotheses would require targeted mutagenesis and comparisons of the wild-type and mutant versions of the transporters, as well as adopting molecular dynamics simulation approaches for these membrane transporters [16].

To investigate conservation of amino acid residues within the selectivity filter and conservation of variant residues between Ni-OsHKT1;5 and Po-OsHKT1;5, we analyzed 213 publicly available amino acid sequences similar to OsHKT1;5 through the ConSurf server [17], including that of HKT1 Na^+^ transporter from Arabidopsis thaliana (GenBank accession Q84TI7). The ConSurf analysis revealed that the Ser-Gly-Gly-Gly motif, forming the selectivity filter of OsHKT1;5 (**Figure 3**) achieved the highest possible conservation (evolutionary conservation score of 9). Conversely, the four Ala140, His184, Asp332 and Val395 amino acid residues that vary between Ni-OsHKT1;5 and Po-OsHKT1;5 (**Figures 2** and **S1)** showed significant variability (evolutionary conservation score of 6).

### Using a multidisciplinary approach to uncover root-to-shoot Na^+^ transfer control in rice upon salt stress

We first suspected the importance of the OsHKT1;5 V395L point mutation based on our previous study, where we compared the salinity response and gene expression profile of four rice lines upon salinity stress [18]. In the previous study, three salt-tolerant lines including Pokkali and two Pokkali-like OsHKT1;5 gene-bearing varieties were compared to IR29, a salt-sensitive cultivar carrying the Nipponbare OsHKT1;5 allele. All the Na^+^ excluding lines carried Val in position 395, while IR29 carried Leu.

We then monitored the shoot Na^+^ concentration and OsHKT1;5 gene root expression level in eight rice lines, including Nipponbare, IR29 and Nona Bokra, while assessing if their respective OsHKT1;5 proteins contained a Leu or Val in position 395. Our results show that the OsHKT1;5 root expression and shoot Na^+^ concentration were inversely correlated within the two respective groups, and that for the same OsHKT1;5 expression levels, the lines containing Leu in position 395 exhibited a higher Na^+^ shoot concentration than those containing Val (**Figure 1e**). Thus, the structural, transcriptomic and physiological data together indicate that the V395L mutation in OsHKT1;5 could be linked with a decreased efficiency of Na^+^ transport. These data also indicate that a structural difference between Leu and Val residues, if critically positioned, can have a decisive impact on Na^+^ transport efficiency, and by extension, on a plant's response to environmental stresses. Alternatively, the decreased efficiency of Na^+^ transport in V395L OsHKT1;5 variants could also be explained by an altered level of proteins in planta but the current lack of available antibody raised against OsHKT proteins stopped us from investigating this hypothesis.

In conclusion, our data could explained the higher efficiency of Na^+^ transport via the Nona Bokra OsHKT1;5 over the Nipponbare-like Koshihikari, as previously reported in the literature [14]. To this end, we find it interesting that the lines containing Leu in position 395 seem to be more sensitive to changes in transcript levels as shown by the trend-line of slopes in **Figure 1e**. Further studies, such as allele characterisation in heterologous systems are required to confirm our findings.

### Sheath-to-blade Na^+^ transfer control in rice upon salt stress cannot be explained by a mechanism similar to that involving HKT1;5 in the roots

**Figure 4:**
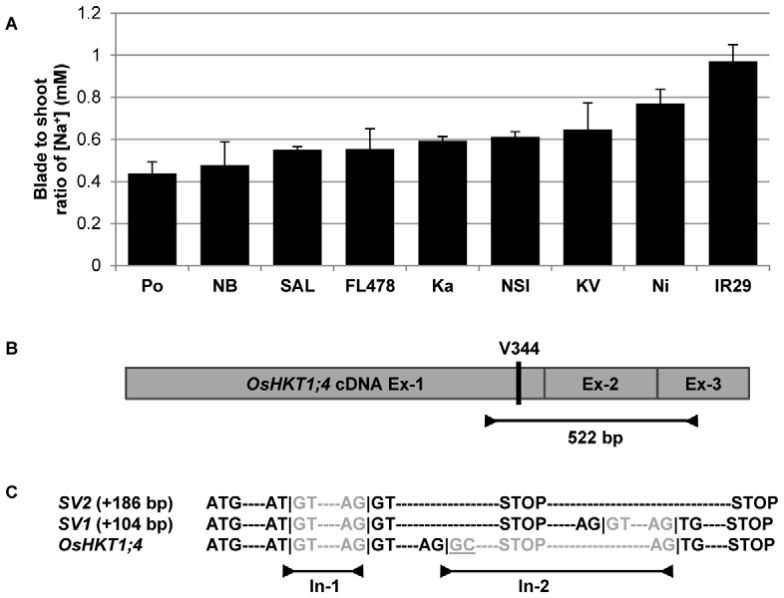
Alternative splicing of the OsHKT1;4 rice gene. (**A**) Flame photometry measurement of the blade-to-shoot ratio of Na^+^ concentrations (+SEM; n = 5) in nine rice lines (The abbreviations Po = Pokkali; NB = Nona Bokra; SAL = SAL208; Ka = Kalurundai; NSI = NSICRC106; KV = Kallurundai Vellai; and Ni = Nipponbare stand for individual varieties; IR29 and FL478 are full names of rice varieties). (**B**) Schematic representation of the OsHKT1;4 cDNA with its three exons (Ex) and the Val residue in position 344. This residue has been identified through the sequencing of the OsHKT1;4 locus genomic DNA in nine rice lines and a 522 bp fragment spanning the three exons in Nipponbare and Pokkali. (**C**) The sequencing of these products shows the presence of two splicing variants of OsHKT1;4: OsHKT1;4-SV1 (+104 bp, i.e. 626 bp total) and OsHKT1;4-SV2 (+186 bp, i.e. 708 bp total). Whereas the first intron (In) is always spliced correctly between a 5′-GT donor site and 3′-AG donor site, the presence of a 5′-GC donor site in the second intron causes alternative splicing [19]. Another intron of a shorter size is spliced in SV1 but not in SV2. Both spliced variants are translated into a truncated OsHKT1;4 protein due to the presence of an in-frame STOP codon at the beginning of the second intron.

We subsequently focused our attention on the Na^+^ movement taking place in the aerial part of the rice plant, and measured Na^+^ concentration under saline conditions in the sheath, blade and whole shoot of nine rice lines: the Pokkali landrace and the eight other rice lines used in the root experiment. Na^+^ concentration patterns varied significantly from line to line, with a high retention of Na^+^ in the leaf sheath of Pokkali and Nona Bokra (blade-to-shoot ratio of [Na^+^]<0.5) to a much lower retention in IR29 (causing a blade-to-shoot ratio of 1) (**Figure 4a**).

Under saline conditions, it appears that OsHKT1;5 is principally expressed in the roots, whereas OsHKT1;4 is sheath-specific (**Figure S2**). To clarify the function of OsHKT1;4 transporters, we constructed a 3D model of Nipponbare OsHKT1;4 (Ni-OsHKT1;4; **Figure S3**), whose sequence was mined from public databases (GenBank accession NP_001053810). The Ni-OsHKT1;4 3D model is similar to the Ni-OsHKT1;5 model, despite the HKT1;4 protein being 47 amino acids shorter than HKT1;5. Due to the length difference in amino acid sequences, the critical OsHKT1;5 residue in position 395 is found in position 344 in OsHKT1;4. However, partial sequencing of the OsHKT1;4 genomic locus in the nine rice lines used in this study showed that all lines share a Val residue in this position (**Figure S4**). Thus, the differences in blade Na^+^ accumulation could not be explained by a mechanism analogous to that involving OsHKT1;5 in the roots.

### Alternative splicing of the OsHKT1;4 gene

Narrowing down our analysis to Pokkali and Nipponbare, we tentatively amplified a unique 522 bp fragment of the OsHKT1;4 transcript spanning all three exons in both lines (**Figure 4b**), but observed the presence of two additional, larger bands for each line after resolving the amplified products (i.e. 3 bands per line). The sequencing of these gene products showed, firstly, that any two bands of similar size were of similar sequence between the two rice lines, and secondly, that all six bands represented OsHKT1;4 gene products. Whereas the lowest observed band of 522 bp was characteristic of the OsHKT1;4 splicing prediction, two other spliced variants of 104 and 186 bp larger than the predicted spliced form were also found, and were designated as OsHKT1;4-SV1 (626 bp; GenBank accession JF920143: Po-OsHKT1;4-SV1; and JF920144: Ni-OsHKT1;4-SV1) and OsHKT1;4-SV2 (708 bp; GenBank accession JF920145: Po-OsHKT1;4-SV2; and JF920146: Ni-OsHKT1;4-SV2), respectively (**Figures 4c** and **S5**).

The OsHKT1;4 first intron was correctly spliced in all three forms, although the GC splicing donor site of the second intron created an instability in splicing. It has been observed that GC-mediated alternative splicing is a naturally occurring phenomenon in rice [19]. Interestingly, both spliced variants would be translated into a truncated OsHKT1;4 protein due to the presence of an in-frame STOP codon in the 5′-end of the second intron. Based on our 3D molecular model, this OsHKT1;4 truncated transporter is predicted to be non-functional (**Figure S3b**), as it lacks a part of a loop and the D4M2 helix in the 4^th^ pseudo-protomer (cf. **Figure S1**). The full-length Po-OsHKT1;4 transcript was sequenced (GenBank accession JF920142) and a single mutation was detected between Ni-OsHKT1;4 and the translated Po-OsHKT1;4 protein sequences (**Figure S6**). We believe that this R110Q mutation should not affect Na^+^ transport rate as it is positioned in a cytoplasmic loop that inter-connects two membrane α-helices of Po-OsHKT1;4.

### Sheath-to-blade Na^+^ transfer control in rice upon salt stress

**Figure 5:**
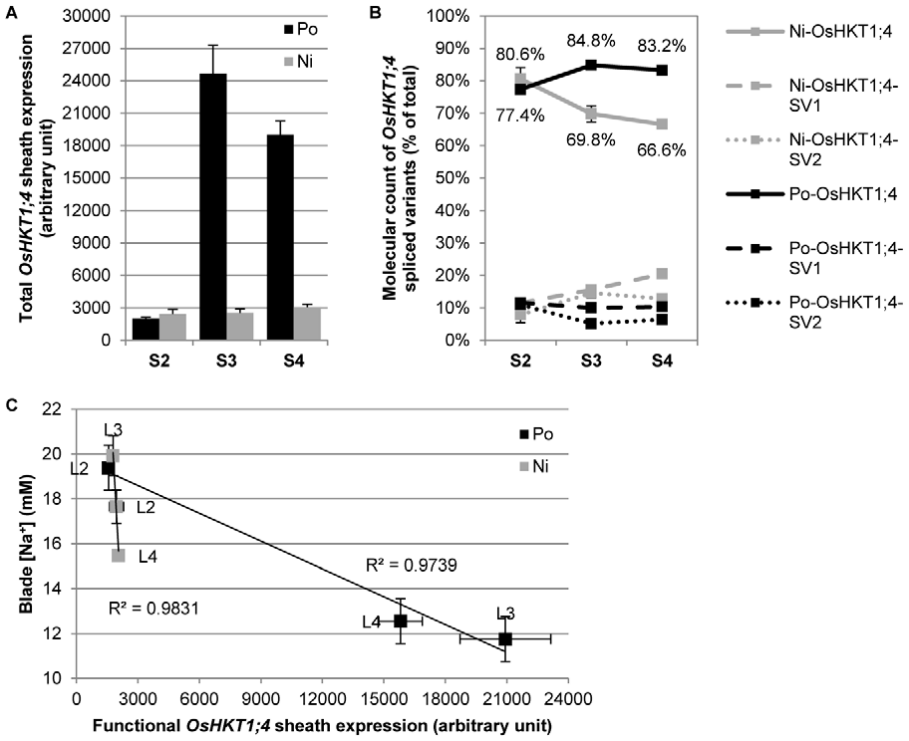
Sheath-to-blade Na^+^ transfer control in rice upon salt stress. (**A**) Total OsHKT1;4 transcript level measured by qRT-PCR (+SEM; n = 3 to 4 replicates per sheath; three technical replicates per biological sample) in individual leaf sheath of Pokkali (Po) and Nipponbare (Ni). Three leaf sheaths were assessed, S2 to S4, from the oldest one to the youngest. (**B**) HPLC quantification of functional OsHKT1;4 transcripts and non-functional spliced variants SV1 and SV2 (+/− SEM; n = 3 to 4 replicates per sheath) in individual leaf sheath of Po and Ni, as a percentage of the total amount of transcripts reported in panel A. Leafs 2 and 3 were fully mature whereas leaf 4, although bigger than leaf 3, was still expending. (**C**) Correlation between functional OsHKT1;4 expression levels in individual sheath (Total OsHKT1;4 transcript level from panel A multiplied by the percentage of functional OsHKT1;4 transcript from panel B; +/− SEM; n = 3 to 4 replicates per sheath) and Na^+^ concentrations in the corresponding blades, measured by flame photometry (+/− SEM; n = 5) in three independent leaves (L2 to L4, from the oldest to the youngest) in Po and Ni.

After no correlation was found between the total sheath or blade tissue Na^+^ concentration and the OsHKT1;4 sheath tissue expression level (**Figure S7**), individual rice leaves were analyzed separately (L2 to L4, from the older to younger leaves). Na^+^ concentration was measured in individual leaf sheaths (S2 to S4) and leaf blades (B2 to B4), and the OsHKT1;4 expression levels in the similar individual leaf sheaths of salt-stressed Pokkali and Nipponbare plants were quantified. All OsHKT1;4 spliced-forms occurring in individual leaf sheaths were analyzed. An HPLC analysis allowed us to quantify the RNA molecular copies for each of the three variants as a percentage of the total OsHKT1;4 transcript levels (**Figure 5b**). Interestingly, Pokkali is able to maintain a much higher functional ratio of OsHKT1;4 transcripts in younger sheath as opposed to Nipponbare. These measurements enabled calculation of the level of correctly spliced OsHKT1;4 transcripts in each individual sheath. It also became apparent that the transcript levels were inversely correlated with the individual leaf blade Na^+^ concentrations in both lines (**Figure 5c**).

Despite the fact that the function of the HKT1;4 spliced variants and associated truncated HKT1;4 proteins remains unknown, it appears that the full length OsHKT1;4 is the key transporter controlling the sheath-to-blade transfer of Na^+^ in rice shoots. It also seems that Pokkali is able to modulate the OsHKT1;4 expression level to cope better with salt stress, which Nipponbare cannot do (**Figure 5a**). Hence, Pokkali is able to both minimize OsHKT1;4 levels in older sheaths to direct and store Na^+^ in senescent photosynthetic tissues, as well as to maximize levels in younger sheaths to preserve the most active energy-producing tissues and, by extension, to increase plant survival under stress.

### A two-staged Na^+^ exclusion model in rice

**Figure 6:**
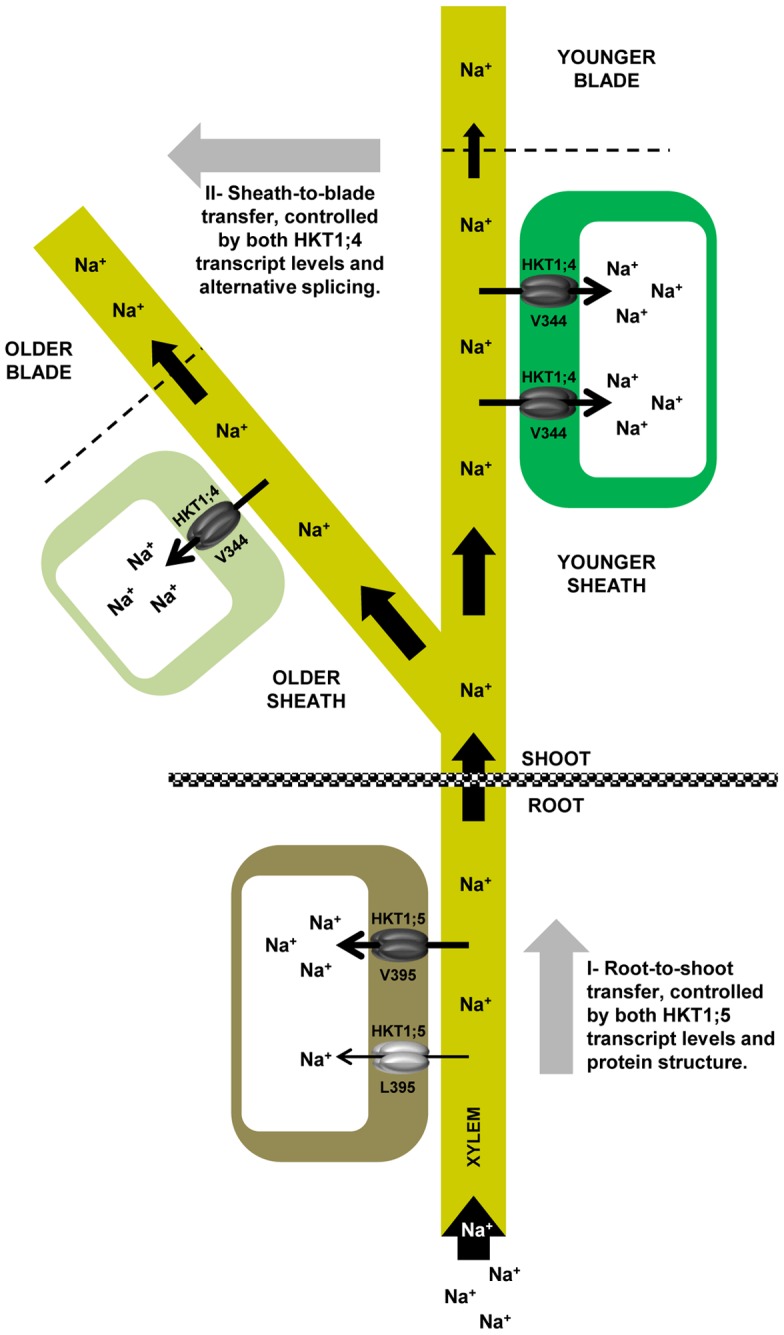
A two-staged Na^+^ exclusion model in rice. Na^+^ ions from the external medium penetrate the root and are transported throughout the plant via xylem vessels. OsHKT1;5 proteins present in xylem parenchymal cells pump Na^+^ ions back into the root to minimize the amount of Na^+^ reaching the shoot, where it is harmful to the plant. This root-to shoot Na^+^ transfer mechanism represents the first stage of a Na^+^ exclusion model in rice, which is controlled by both OsHKT1;5 transcript levels and structural determinants of the OsHKT1;5 protein. High excluding lines carry a Val instead of a Leu in position 395, and this protein variation mediates a faster Na^+^ transport rate. The remaining Na^+^ ions that arrive into the shoot are diverted into different leaves. There, OsHKT1;4 proteins load the sheath tissues with Na^+^ ions before they can reach the photosynthetic part of the shoot, i.e. the blades. This sheath-to-blade Na^+^ transfer mechanism represents the second stage of the Na^+^ exclusion model in rice. Na^+^ excluding lines maximize this second dimension by firstly, having higher OsHKT1;4 expression levels in younger sheaths to protect the more energy-producing young blades and secondly, by controlling the ratio of spliced transcripts in favor of transcripts translated into functional proteins. Older leaves, with lower levels of the OsHKT1;4 proteins in the sheath, let Na^+^ go through to the senescing leaf blades, where Na^+^ can safely be stored and does no harm the plant.

To summarize, through 3D comparative modeling of two rice HKT transporters and a discovery of alternative splicing of OsHKT1;4 transcripts, we were able to suggest a two-staged Na^+^ exclusion model in rice (**Figure 6**). This model is composed first of a vertical axis from root-to-shoot of the plant that is controlled by both OsHKT1;5 expression and the specific structural determinants of OsHKT1;5 transporters. The second component is represented by a horizontal axis in the shoot system that advances from older leaves to younger leaves and which is controlled by both OsHKT1;4 expression and splicing (**Figure 6**).

## Materials and Methods

### Plant material, growth and salinity assay

All rice seeds were obtained from IRRI in the Philippines. Dehusked seeds were surface-sterilized in 1% (w/v) sodium hypochlorite and rinsed several times before being germinated in deep cell culture dishes in a growth chamber (28/24°C day/night; 12-h photoperiod; 80% humidity). After five days, seedlings were transplanted to a 10-L or 120-L hydroponic tank (depending on the size of the experiment) filled with nutrient solution [5 mM NH_4_NO_3_; 5 mM KNO_3_; 2 mM Ca(NO_3_)_2_; 2 mM MgSO_4_; 0.1 mM KH_2_PO_4_; 0.05 mM NaFe(III)EDTA; 50 µM H_3_BO_3_; 5 µM MnCl_2_; 5 µM ZnSO_4_; 0.5 µM CuSO_4_; and 0.1 µM Na_2_MoO_3_; pH 5.5]. The solution was changed after seven days, and a mild salt stress was applied in two increments: 40 mM NaCl and 0.64 mM CaCl_2_ for four days, followed by 70 mM NaCl and 1 mM CaCl_2_ for five more days. CaCl_2_ was added to the solution to maintain a constant Ca^2+^ activity of 1 mM throughout the experiment. Selected tissues were harvested three weeks after germination and their Na^+^ content measured with a flame photometer (Sherwood Scientific Ltd, Cambridge, UK).

### Radioactive ^22^Na^+^ fluxes

Experiments were conducted using 14-day-old intact plants grown as described above. The plants were salt-treated for three days prior to the experiments with 30 mM NaCl and 0.5 mM CaCl_2_ to maintain a constant Ca^2+^ activity of 1 mM. The roots of intact plants were blotted to remove adhering water, then placed in 50 mL of influx solution (30 mM NaCl, 0.5 mM KCl and 0.1 mM CaCl_2_ activity) labelled with radioactive ^22^Na^+^ (0.05 µCi/ml) (Amersham/GE Healthcare, Sydney, Australia). Experiments were conducted on gently rotating shakers (35 rpm) to keep solutions aerated and reduce boundary layer effects. Following influx treatment (2 min), intact plants were removed from the influx solution and extra influx solution was allowed to drip off roots. Roots were rinsed rapidly at room temperature with a rinse solution (30 mM NaCl, 0.5 mM KCl and 10 mM CaCl_2_) to displace apoplastically bound ^22^Na^+^, then excised below the seed and rested in tea strainers through two successive rinses in 500 ml of ice-cold (4°C) rinse solution (2 min+2 min) on gently rotating shakers (35 rpm). Roots were blotted dry with paper towels and immediately weighed in six ml scintillation vials (PerkinElmer, Melbourne, Australia). Shoots were separated into sheath and blade tissue and weighed in scintillation vials. Four ml of an Ecolume scintillation fluid (MP Biomedicals, Sydney, Australia) was added to each vial and vial contents were mixed and measured using a liquid scintillation counter (Beckman Coulter LS6500, Gladesville, Australia). Immediately prior to and immediately following each experiment, three 20 µL samples of the influx solution were placed into scintillation vials, and scintillation fluid was added and mixed to enable calculation of specific activity in each influx solution. This allowed conversion of radioactivity in tissues to amounts of chemical Na^+^, and thus a conversion to a Na^+^ flux. Blank vials were also measured to allow subtraction of background radiation from the measured values.

### RNA extraction and qRT-PCR

RNA was purified from plant material with TRIzol (Invitrogen) and treated with rDNase I using a DNA-free kit (Ambion), before cDNA synthesis was performed on 1 µg of RNA with a 19-mer polyT primer and a SuperScript III reverse transcriptase kit (Invitrogen), according to the manufacturers' instructions. Real-time quantitative PCR (qRT-PCR) was carried out as previously described [20]. The dataset was normalised with three control genes for the root (Actin, GAP and ELF) and four control genes for the sheath (same as in root plus tubulin) [18].

### Quantification of spliced forms

A total of 60 µL of a RT-PCR product spanning the OsHKT1;4 second intron and amplified with the primers CCGAGAGAAGAAAGCTCAAAGAAGAC (forward) and AGAGATGGTCTGGATTGATCTGTCTACT (reverse) were used to quantitate the three spliced forms in each tissue by HPLC. The mass of each spliced form present in each PCR reaction was determined by integrating the HPLC peak area of the spliced form and using a plasmid digest of known concentration to convert peak area to mass (ng) [20], [21]. The masses were converted first into a relative molar amount and then to a percentage of the total of all spliced forms. The averages of these percentages were calculated for each cultivar, sheath/leaf number and spliced form of OsHKT1;4.

### Construction of 3D models of Ni-HKT1;5, Po-HKT1;5, HKT1;4 and spliced HKT1;4

The most suitable template for the OsHKT1 proteins, identified via 3D-PSSM [22] and the Structure Prediction Meta-Server [23] was a TrkH K^+^ transporter [Protein Data Bank (PDB), accession number 3PJZ] from Vibrio parahaemolyticus [15]. This transporter occurs as a homodimer in-solution, consists of five domains and adopts a pseudo-fourfold symmetry. TrkH was crystallized in the presence of 150 mM KCl and contains K^+^ ions in its central pore^10^. The sequences of 3PJZ and Ni-OsHKT1;5, Po-OsHKT1;5, Ni-OsHKT1;4 and spliced Ni-OsHKT1;4 were aligned via LOMETS (LOcal MEta-Threading-Server) [24] and several alignment possibilities obtained through PSIPRED [25] and PROLALS3d [26] were tested for the positions of secondary structures. The latter were further checked by a hydrophobic cluster analysis (HCA) [27] (**Figure S1**). The aligned sequences were used as input parameters to generate the 3D models of Ni-OsHKT1;5 (554 residues), Po-OsHKT1;5 (554), Ni-OsHKT1;4 (500) and its spliced form (450) in complex with a Na^+^ ion using Modeller 9v8 [28] on a Linux station, running Fedora 12 operating system. The final 3D molecular model of each OsHKT transporter was selected from 50 models. The models with the lowest value of the Modeller 9v8 objective function and the most favourable DOPE energy scoring parameters were chosen for optimisation with Tripos force field within the Sybyl 8.0 suite of programs (Tripos International). A Ramachandran plot of 3PJZ (455 residues) and the Ni-OsHKT1;5 (475), Po-OsHKT1;5 (476), Ni-OsHKT1;4 (500) and its spliced form (450) optimized models indicated that 99% (390), 99.2% (476), 99% (437), 98.6% (437), 99.5% (398) residues were in the most favoured, additionally allowed and generously allowed regions, when excluding glycine and proline residues. The overall G-factors (estimates of stereochemical parameters) evaluated by PROCHECK [29], were −0.1 for all structures. The Z-score values deduced from ProSa2003 [30] reflecting combined statistical potential energy were −8.8, −5.0, −5.3, −5.7 and −4.6 for 3PJZ, Ni-OsHKT1;5, Po-OsHKT1;5, Ni-OsHKT1;4 and Ni-OsHKT1;4 spliced, respectively. The root mean square deviation values, between 3PJZ and Ni-OsHKT1;5 (374 residues), Po-OsHKT1;5 (392), Ni-OsHKT1;4 (387) and Ni-OsHKT1;4 spliced (356), determined with ‘iterative magic fit’ algorithm (that aligns protein structurally) in Deep-View (http://spdbv.vital-it.ch/) were 0.76 Å, 0.92 Å, 0.80 Å and 0.79 Å, respectively in Cα positions.

## Supporting Information

Figure S1 **Hydrophobic cluster analysis of rice Ni-HKT1;5 and TrkH.** Positions of membrane helices are shown by blue arrows, and four insertions 1-4 in Ni-HKT1;5 are marked by black lines. IL in TrkH is intracellular loop. The positions of pore helices (PH) and selectivity filters (SF) containing Gly-Gly-Gly-Gly and Ser-Gly-Gly-Gly signatures are shown in magenta and white arrows in the TrkH and Ni-HKT1;5 plots. The proline residues are shown as red stars, glycine residues as black diamonds, serine residues are empty squares and threonine residues are shown as squares containing with a black dot in the centre. Negatively charged residues are coloured in red and positively charged residues are in blue. Other residues are shown by their single amino acid letter codes. The amino acid numbers are shown, and read from the top to the bottom of the plots (in duplicate) in a left to right direction. The variant residues between Po-HKT1;5 and Ni-HKT1;5 sequences (A140P, H184R, D332H and V395L) are enclosed in purple circles and are indicated by enlarged types.
(EPS)

Figure S2 ***OsHKT1;4*** **and** ***OsHKT1;5*** **expression in rice.** (**A**) *OsHKT1;4* and *OsHKT1;5* transcript levels measured by qRT-PCR in the root of 8 rice lines salt treated as per described in the methods section (3 biological reps per line/tissue; 3 technical reps per biological sample). (**B**) Similar for sheath tissue. (C) *OsHKT1;4* root transcript levels measured by qRT-PCR in 4 salt treated rice lines. The biological material used for this panel is the same as the one described in Cotsaftis *et al.* (2011). Overall, it appears that in rice and under saline conditions, *OsHKT1;4* is a sheath specific gene whereas *OsHKT1;5* is principally expressed in the roots. Root and sheath data, or root data from different experiments are not comparable as the different dataset were normalized separately. However, an expression level 500 (arbitrary unit) is likely to represent background noise and is not representative of an actual expression level. The abbreviations Po = Pokkali; NB = Nona Bokra; Ni = Nipponbare; KV = Kallurundai Vellai; Ka = Kalurundai; NSI = NSICRC106; and SAL = SAL208 stand for individual varieties, variously represented through the panels A-C. IR29 and FL478 are full names of rice varieties.
(PDF)

Figure S3 **Molecular models of the full-length and spliced HKT1;4 transporters from rice.** (**A**) A cartoon representation of full-length HKT1;4, illustrating its overall fold. (**B**) A cartoon representation of spliced HKT1;4, illustrating the absence of a part of a loop and the D4M2 helix in the 4th pseudo-protomer (cf. **Figure S1**). The COOH-terminal residue of spliced HKT1;4 is indicated by a small arrow. The positions of Na+ ions (purple spheres) are shown near the selectiv-ity filters. Black lines indicate the Ser-Gly-Gly-Gly signatures in both HKT1;4 transporters. The entry into the pores of transporters is indicated by black arrows.
(EPS)

Figure S4 **Sequence comparison of** ***OsHKT1;4*** **genomic DNA fragments across several rice lines.** Two regions of the *OsHKT1;4* gene were sequenced from genomic DNA in nine rice lines. The first region (∼300 bp) covers the 3′-end of the first exon up until the 5′-end of the first intron and the second region (∼500 bp) from the start of the second exon until the 5′-end of the third exon. The sequences obtained were aligned with the Ni-*OsHKT1;4* locus genomic sequence mined from public databases. No differences were observed in any of the lines analyzed besides a point mutation in the second intron of Nipponbare which may represent a *japonica* v. *indica* single nucleotide polymorphism. Sequence alignment was performed using Align X (Invitrogen). Homologous sequences are highlighted in yellow or blue. The conserved codon encoding a Val residue in position 344 of the OsHKT1;4 protein is highlighted in green. The abbreviations Po = Pokkali; NB = Nona Bokra; Ni = Nipponbare; KV = Kallurundai Vellai; Ka = Kalurundai; NSI = NSICRC106; and SAL = SAL208 stand for individual varieties, whereas IR29 and FL478 are full names of rice varieties.
(PDF)

Figure S5 **Sequences of** ***OsHKT1;4*** **splice variants.** A predicted 522 bp fragment spanning over the *OsHKT1;4* transcript three exons was amplified by RT-PCR from RNA purified from salt treated Nipponbare and Pokkali sheath tissue. Three amplification products were recovered from each line: the predicted 522 bp fragment (HKT1;4) and two other fragments of superior sizes (HKT1;4-SV1 and HKT1;4-SV2). No sequence variation was observed between the fragments of similar size across the two lines (data not shown). Here, an alignment of the three splice forms is presented against the genomic sequence of the *OsHKT1;4* gene (Genomic). The increased length of both splice variants is due to alternative splicing of the second intron. Whereas *SV1* is splicing a second shorter intron, only one intron, the first, is spliced in *SV2*. Both variants are translated into an identical truncated protein due to the presence of an in frame STOP codon in the 5′-end of *OsHKT1;4* second intron. Sequence alignment was performed using Align X (Invitrogen). Homologous sequences are highlighted in yellow or blue. The full length second intron is highlighted in blue. The conserved codon encoding a Val residue in position 344 of the *OsHKT1;4* protein is highlighted in green. The in frame STOP codon in the second intron is highlighted in purple. The RT-PCR primers are highlighted in grey. The sequences of the primers used to perform the HPLC molecular count ratio between the three splice forms are underlined in black.
(PDF)

Figure S6
**OsHKT1;4 protein sequence comparison.** The Pokkali (Po) *OsHKT1;4* transcript encoding a functional protein was fully sequenced and its nucleotide sequence translated into an amino acid sequence. Po-*OsHKT1;4* encodes for a 500 amino acid protein which share 99.8% homology with the OsHKT1;4 protein from Nipponbare (Ni). The sole difference between the two functional OsHKT1;4 proteins is a R to Q substitution in position 110 in Pokkali. The structural modeling of the OsHKT1;4 protein predicts that this substitution is localized in the cytoplasm-exposed loop inter-connecting membrane 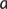-helices between the first two domains of the protein and thus does not affect Na+ transport rate. Ni-OsHKT1;4 and Po-OsHKT1;4 were aligned against a predicted truncated protein generated by the splice variants. This predicted splice variant protein is 47 amino acid residues shorter and differs from the full-length OsHKT1;4 protein in its 19 C-terminal residues. The protein sequences were aligned using Align X (Invitrogen). Homologous regions are highlighted in yellow or blue. The conserved Val residue in position 344 is highlighted in green.(PDF)

Figure S7 **Correlation between** ***OsHKT1;4*** **sheath expression level and Na+ tissue concentration.** (**A**) Correlation between sheath *OsHKT1;4* expression level measured by qRT-PCR (3 biological reps per line; 3 technical reps per biological sample) and sheath Na+ concentration measured by flame photometry (n = 5) in 8 rice lines salt treated as per described in the methods section. (**B**) Similar correlation with blade Na+ concentration. No correlation were observed. The abbreviations NB = Nona Bokra; Ni = Nipponbare; KV = Kallurundai Vellai; Ka = Kalurundai; NSI = NSICRC106; and SAL = SAL208 stand for individual varieties, whereas IR29 and FL478 are full names of rice varieties.
(PDF)
