# Supplementary material for: A Two-Staged Model of Na+ Exclusion in Rice Explained by 3D Modeling of HKT Transporters and Alternative Splicing
Source: PLoS One. 2012 Jul 11;7(7):e39865. doi: 10.1371/journal.pone.0039865 (PMC3394774; doi:10.1371/journal.pone.0039865)
Supplement: Figure S2 — OsHKT1;4 and OsHKT1;5 expression in rice. (A) OsHKT1;4 and OsHKT1;5 transcript levels measured by qRT-PCR in the root of 8 rice lines salt treated as per described in the methods section (3 biological reps per line/tissue; 3 technical reps per biological sample). (B) Similar for sheath tissue. (C) OsHKT1;4 root transcript levels measured by qRT-PCR in 4 salt treated rice lines. The biological material used for this panel is the same as the one described in Cotsaftis et al. (2011). Overall, it appears that in rice and under saline conditions, OsHKT1;4 is a sheath specific gene whereas OsHKT1;5 is principally expressed in the roots. Root and sheath data, or root data from different experiments are not comparable as the different dataset were normalized separately. However, an expression level 500 (arbitrary unit) is likely to represent background noise and is not representative of an actual expression level. The abbreviations Po = Pokkali; NB = Nona Bokra; Ni = Nipponbare; KV = Kallurundai Vellai; Ka = Kalurundai; NSI = NSICRC106; and SAL = SAL208 stand for individual varieties, variously represented through the panels A-C. IR29 and FL478 are full names of rice varieties. (PDF) [file pone.0039865.s002.pdf]

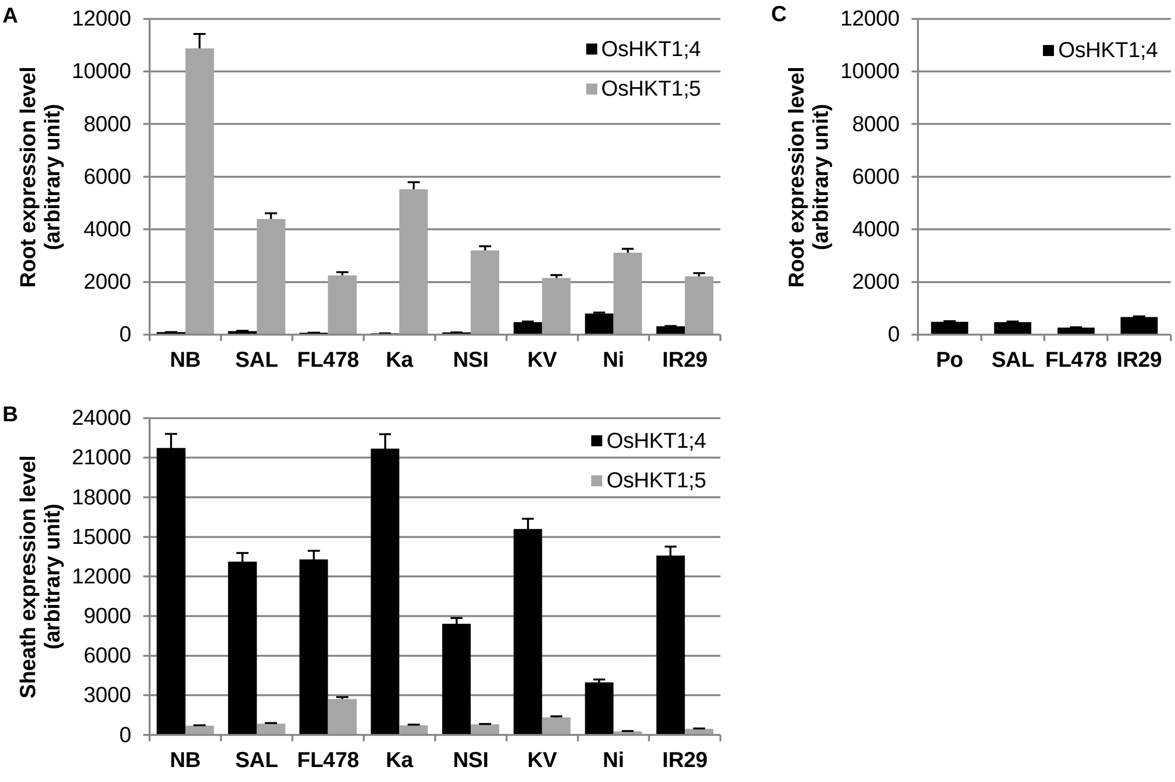

Figure S2. *OsHKT1;4* and *OsHKT1;5* expression in rice. (**A**) *OsHKT1;4* and *OsHKT1;5* transcript levels measured by qRT-PCR in the root of 8 rice lines salt treated as per described in the methods section (3 biological reps per line/tissue; 3 technical reps per biological sample). (**B**) Similar for sheath tissue. (**C**) *OsHKT1;4* root transcript levels measured by qRT-PCR in 4 salt treated rice lines. The biological material used for this panel is the same as the one described in Cotsaftis *et al*. (2011). Overall, it appears that in rice and under saline conditions, *OsHKT1;4* is a sheath specific gene whereas *OsHKT1;5* is principally expressed in the roots. Root and sheath data, or root data from different experiments are not comparable as the different dataset were normalized separately. However, an expression level $<$ 500 (arbitrary unit) is likely to represent background noise and is not representative of an actual expression level. The abbreviations Po = Pokkali; NB = Nona Bokra; Ni = Nipponbare; KV = Kallurundai Vellai; Ka = Kalurundai; NSI = NSICRC106; and SAL = SAL208 stand for individual varieties, variously represented through the panels A-C. IR29 and FL478 are full names of rice varieties.
