# Supplementary material for: A Two-Staged Model of Na+ Exclusion in Rice Explained by 3D Modeling of HKT Transporters and Alternative Splicing
Source: PLoS One. 2012 Jul 11;7(7):e39865. doi: 10.1371/journal.pone.0039865 (PMC3394774; doi:10.1371/journal.pone.0039865)
Supplement: Figure S4 — Sequence comparison of OsHKT1;4 genomic DNA fragments across several rice lines. Two regions of the OsHKT1;4 gene were sequenced from genomic DNA in nine rice lines. The first region (∼300 bp) covers the 3′-end of the first exon up until the 5′-end of the first intron and the second region (∼500 bp) from the start of the second exon until the 5′-end of the third exon. The sequences obtained were aligned with the Ni-OsHKT1;4 locus genomic sequence mined from public databases. No differences were observed in any of the lines analyzed besides a point mutation in the second intron of Nipponbare which may represent a japonica v. indica single nucleotide polymorphism. Sequence alignment was performed using Align X (Invitrogen). Homologous sequences are highlighted in yellow or blue. The conserved codon encoding a Val residue in position 344 of the OsHKT1;4 protein is highlighted in green. The abbreviations Po = Pokkali; NB = Nona Bokra; Ni = Nipponbare; KV = Kallurundai Vellai; Ka = Kalurundai; NSI = NSICRC106; and SAL = SAL208 stand for individual varieties, whereas IR29 and FL478 are full names of rice varieties. (PDF) [file pone.0039865.s004.pdf]

```
                        1401                                              1450
          FL478      (1) -------ATGCCGGCGCGGCGGTGCTGGATGCTGGCGGCGACGGTGGCGG
           IR29      (1) -------ATGCCGGCGCGGCGGTGCTGGATGCTGGCGGCGACGGTGGCGG
             Ka      (1) -------ATGCCGGCGCGGCGGTGCTGGATGCTGGCGGCGACGGTGGCGG
             KV      (1) -------ATGCCGGCGCGGCGGTGCTGGATGCTGGCGGCGACGGTGGCGG
             NB      (1) -------ATGCCGGCGCGGCGGTGCTGGATGCTGGCGGCGACGGTGGCGG
             Ni      (1) -------ATGCCGGCGCGGCGGTGCTGGATGCTGGCGGCGACGGTGGCGG
            NSI      (1) -------ATGCCGGCGCGGCGGTGCTGGATGCTGGCGGCGACGGTGGCGG
             Po      (1) -------ATGCCGGCGCGGCGGTGCTGGATGCTGGCGGCGACGGTGGCGG
            SAL      (1) -------ATGCCGGCGCGGCGGTGCTGGATGCTGGCGGCGACGGTGGCGG
    Ni-HKT1;4 Ex1  (846) GCACCTGATGCCGGCGCGGCGGTGCTGGATGCTGGCGGCGACGGTGGCGG
   Ni-HKT1;4 gDNA (1401) GCACCTGATGCCGGCGCGGCGGTGCTGGATGCTGGCGGCGACGGTGGCGG

                        1451                                              1500
          FL478     (42) CGTTCGTCGCCGTGCTGATGGCGCTGGTGTGCGGCATGGAGTGGGGCGGG
           IR29     (42) CGTTCGTCGCCGTGCTGATGGCGCTGGTGTGCGGCATGGAGTGGGGCGGG
             Ka     (42) CGTTCGTCGCCGTGCTGATGGCGCTGGTGTGCGGCATGGAGTGGGGCGGG
             KV     (42) CGTTCGTCGCCGTGCTGATGGCGCTGGTGTGCGGCATGGAGTGGGGCGGG
             NB     (42) CGTTCGTCGCCGTGCTGATGGCGCTGGTGTGCGGCATGGAGTGGGGCGGG
             Ni     (42) CGTTCGTCGCCGTGCTGATGGCGCTGGTGTGCGGCATGGAGTGGGGCGGG
            NSI     (44) CGTTCGTCGCCGTGCTGATGGCGCTGGTGTGCGGCATGGAGTGGGGCGGG
             Po     (44) CGTTCGTCGCCGTGCTGATGGCGCTGGTGTGCGGCATGGAGTGGGGCGGG
            SAL     (44) CGTTCGTCGCCGTGCTGATGGCGCTGGTGTGCGGCATGGAGTGGGGCGGG
    Ni-HKT1;4 Ex1  (896) CGTTCGTCGCCGTGCTGATGGCGCTGGTGTGCGGCATGGAGTGGGGCGGG
   Ni-HKT1;4 gDNA (1451) CGTTCGTCGCCGTGCTGATGGCGCTGGTGTGCGGCATGGAGTGGGGCGGG

                        1501                                              1550
          FL478     (92) GCGCTGCAGGGGATGAGCCCGTGGGAGAAGGTGGTGAACGCGCTGTTCCT
           IR29     (92) GCGCTGCAGGGGATGAGCCCGTGGGAGAAGGTGGTGAACGCGCTGTTCCT
             Ka     (92) GCGCTGCAGGGGATGAGCCCGTGGGAGAAGGTGGTGAACGCGCTGTTCCT
             KV     (92) GCGCTGCAGGGGATGAGCCCGTGGGAGAAGGTGGTGAACGCGCTGTTCCT
             NB     (92) GCGCTGCAGGGGATGAGCCCGTGGGAGAAGGTGGTGAACGCGCTGTTCCT
             Ni     (92) GCGCTGCAGGGGATGAGCCCGTGGGAGAAGGTGGTGAACGCGCTGTTCCT
            NSI     (94) GCGCTGCAGGGGATGAGCCCGTGGGAGAAGGTGGTGAACGCGCTGTTCCT
             Po     (94) GCGCTGCAGGGGATGAGCCCGTGGGAGAAGGTGGTGAACGCGCTGTTCCT
            SAL     (94) GCGCTGCAGGGGATGAGCCCGTGGGAGAAGGTGGTGAACGCGCTGTTCCT
    Ni-HKT1;4 Ex1  (946) GCGCTGCAGGGGATGAGCCCGTGGGAGAAGGTGGTGAACGCGCTGTTCCT
   Ni-HKT1;4 gDNA (1501) GCGCTGCAGGGGATGAGCCCGTGGGAGAAGGTGGTGAACGCGCTGTTCCT

                        1551                                              1600
          FL478    (142) CGCCGTGAACGCCCGGCACACCGGCGAGTCCACCGTCGACCTCTCCATCC
           IR29    (142) CGCCGTGAACGCCCGGCACACCGGCGAGTCCACCGTCGACCTCTCCATCC
             Ka    (142) CGCCGTGAACGCCCGGCACACCGGCGAGTCCACCGTCGACCTCTCCATCC
             KV    (142) CGCCGTGAACGCCCGGCACACCGGCGAGTCCACCGTCGACCTCTCCATCC
             NB    (142) CGCCGTGAACGCCCGGCACACCGGCGAGTCCACCGTCGACCTCTCCATCC
             Ni    (142) CGCCGTGAACGCCCGGCACACCGGCGAGTCCACCGTCGACCTCTCCATCC
            NSI    (144) CGCCGTGAACGCCCGGCACACCGGCGAGTCCACCGTCGACCTCTCCATCC
             Po    (144) CGCCGTGAACGCCCGGCACACCGGCGAGTCCACCGTCGACCTCTCCATCC
            SAL    (144) CGCCGTGAACGCCCGGCACACCGGCGAGTCCACCGTCGACCTCTCCATCC
    Ni-HKT1;4 Ex1  (996) CGCCGTGAACGCCCGGCACACCGGCGAGTCCACCGTCGACCTCTCCATCC
   Ni-HKT1;4 gDNA (1551) CGCCGTGAACGCCCGGCACACCGGCGAGTCCACCGTCGACCTCTCCATCC

                        1601                                              1650
          FL478    (192) TCGCGCCGGCCATCCTCGTGCTCTTCGTCCTCATGATGTGAGTGCTCTCT
           IR29    (192) TCGCGCCGGCCATCCTCGTGCTCTTCGTCCTCATGATGTGAGTGCTCTCT
             Ka    (192) TCGCGCCGGCCATCCTCGTGCTCTTCGTCCTCATGATGTGAGTGCTCTCT
             KV    (192) TCGCGCCGGCCATCCTCGTGCTCTTCGTCCTCATGATGTGAGTGCTCTCT
             NB    (192) TCGCGCCGGCCATCCTCGTGCTCTTCGTCCTCATGATGTGAGTGCTCTCT
             Ni    (192) TCGCGCCGGCCATCCTCGTGCTCTTCGTCCTCATGATGTGAGTGCTCTCT
            NSI    (194) TCGCGCCGGCCATCCTCGTGCTCTTCGTCCTCATGATGTGAGTGCTCTCT
             Po    (194) TCGCGCCGGCCATCCTCGTGCTCTTCGTCCTCATGATGTGAGTGCTCTCT
            SAL    (194) TCGCGCCGGCCATCCTCGTGCTCTTCGTCCTCATGATGTGAGTGCTCTCT
    Ni-HKT1;4 Ex1 (1046) TCGCGCCGGCCATCCTCGTGCTCTTCGTCCTCATGAT-------------
   Ni-HKT1;4 gDNA (1601) TCGCGCCGGCCATCCTCGTGCTCTTCGTCCTCATGATGTGAGTGCTCTCT
```

```
                         1651                                             1700
         FL478    (242) GTTTCTCTCTCTGACCTAAGCTCTCTCAGG--------------------
          IR29    (242) GTTTCTCTCTCTGACCTAAGCTCTCTCAGG--------------------
            Ka    (242) GTTTCTCTCTCTGACCTAAGCTCTCTCAGG--------------------
            KV    (242) GTTTCTCTCTCTGACCTAAGCTCTCTCAGG--------------------
            NB    (242) GTTTCTCTCTCTGACCTAAGCTCTCTCAGG--------------------
            Ni    (242) GTTTCTCTCTCTGACCTAAGCTCTCTCAGG--------------------
           NSI    (244) GTTTCTCTCTCTGACCTAAGCTCTCTCAGG--------------------
            Po    (244) GTTTCTCTCTCTGACCTAAGCTCTCTCAGG--------------------
           SAL    (244) GTTTCTCTCTCTGACCTAAGCTCTCTCAGG--------------------
  Ni-HKT1;4 Ex1  (1084) --------------------------------------------------
 Ni-HKT1;4 gDNA  (1651) GTTTCTCTCTCTGACCTAAGCTCTCTCAGGGTCTCAGCTCTTCAACCTAG

                         4301                                             4350
         FL478      (1) ----------------------------------GTATCTACCTCCGTAC
          IR29      (1) ----------------------------------GTATCTACCTCCGTAC
            Ka      (1) ----------------------------------GTATCTACCTCCGTAC
            KV      (1) ----------------------------------GTATCTACCTCCGTAC
            NB      (1) ----------------------------------GTATCTACCTCCGTAC
            Ni      (1) ----------------------------------GTATCTACCTCCGTAC
           NSI      (1) ----------------------------------GTATCTACCTCCGTAC
            Po      (1) ----------------------------------GTATCTACCTCCGTAC
           SAL      (1) ----------------------------------GTATCTACCTCCGTAC
  Ni-HKT1;4 Ex2     (1) ----------------------------------GTATCTACCTCCGTAC
 Ni-HKT1;4 gDNA  (4301) TGTTGTCTCATATTCTGCTTCATCTTGCCTCCAGGTATCTACCTCCGTAC

                         4351                                             4400
         FL478      (6) ACGACGTGGTTCCCATTTGAAGAGAATTCCACTACTAAGGATAGTAATGC
          IR29      (6) ACGACGTGGTTCCCATTTGAAGAGAATTCCACTACTAAGGATAGTAATGC
            Ka      (6) ACGACGTGGTTCCCATTTGAAGAGAATTCCACTACTAAGGATAGTAATGC
            KV      (6) ACGACGTGGTTCCCATTTGAAGAGAATTCCACTACTAAGGATAGTAATGC
            NB      (6) ACGACGTGGTTCCCATTTGAAGAGAATTCCACTACTAAGGATAGTAATGC
            Ni      (6) ACGACGTGGTTCCCATTTGAAGAGAATTCCACTACTAAGGATAGTAATGC
           NSI      (6) ACGACGTGGTTCCCATTTGAAGAGAATTCCACTACTAAGGATAGTAATGC
            Po      (6) ACGACGTGGTTCCCATTTGAAGAGAATTCCACTACTAAGGATAGTAATGC
           SAL      (6) ACGACGTGGTTCCCATTTGAAGAGAATTCCACTACTAAGGATAGTAATGC
  Ni-HKT1;4 Ex2    (16) ACGACGTGGTTCCCATTTGAAGAGAATTCCACTACTAAGGATAGTAATGC
 Ni-HKT1;4 gDNA  (4351) ACGACGTGGTTCCCATTTGAAGAGAATTCCACTACTAAGGATAGTAATGC

                         4401                                             4450
         FL478     (56) AGAGAACCAGGGAATCAGACTGCTCGAGAGTACACTTTTGTCACAACTCT
          IR29     (56) AGAGAACCAGGGAATCAGACTGCTCGAGAGTACACTTTTGTCACAACTCT
            Ka     (56) AGAGAACCAGGGAATCAGACTGCTCGAGAGTACACTTTTGTCACAACTCT
            KV     (56) AGAGAACCAGGGAATCAGACTGCTCGAGAGTACACTTTTGTCACAACTCT
            NB     (56) AGAGAACCAGGGAATCAGACTGCTCGAGAGTACACTTTTGTCACAACTCT
            Ni     (56) AGAGAACCAGGGAATCAGACTGCTCGAGAGTACACTTTTGTCACAACTCT
           NSI     (56) AGAGAACCAGGGAATCAGACTGCTCGAGAGTACACTTTTGTCACAACTCT
            Po     (56) AGAGAACCAGGGAATCAGACTGCTCGAGAGTACACTTTTGTCACAACTCT
           SAL     (56) AGAGAACCAGGGAATCAGACTGCTCGAGAGTACACTTTTGTCACAACTCT
  Ni-HKT1;4 Ex2    (66) AGAGAACCAGGGAATCAGACTGCTCGAGAGTACACTTTTGTCACAACTCT
 Ni-HKT1;4 gDNA  (4401) AGAGAACCAGGGAATCAGACTGCTCGAGAGTACACTTTTGTCACAACTCT

                         4451                                             4500
         FL478    (106) CCTACCTGACCATCTTTGTCATTGCCATCTGCATCACCGAGAGAAGAAAG
          IR29    (106) CCTACCTGACCATCTTTGTCATTGCCATCTGCATCACCGAGAGAAGAAAG
            Ka    (106) CCTACCTGACCATCTTTGTCATTGCCATCTGCATCACCGAGAGAAGAAAG
            KV    (106) CCTACCTGACCATCTTTGTCATTGCCATCTGCATCACCGAGAGAAGAAAG
            NB    (106) CCTACCTGACCATCTTTGTCATTGCCATCTGCATCACCGAGAGAAGAAAG
            Ni    (106) CCTACCTGACCATCTTTGTCATTGCCATCTGCATCACCGAGAGAAGAAAG
           NSI    (106) CCTACCTGACCATCTTTGTCATTGCCATCTGCATCACCGAGAGAAGAAAG
            Po    (106) CCTACCTGACCATCTTTGTCATTGCCATCTGCATCACCGAGAGAAGAAAG
           SAL    (106) CCTACCTGACCATCTTTGTCATTGCCATCTGCATCACCGAGAGAAGAAAG
  Ni-HKT1;4 Ex2   (116) CCTACCTGACCATCTTTGTCATTGCCATCTGCATCACCGAGAGAAGAAAG
 Ni-HKT1;4 gDNA  (4451) CCTACCTGACCATCTTTGTCATTGCCATCTGCATCACCGAGAGAAGAAAG
```

```
                              4501                                             4550
           FL478    (156)  CTCAAAGAAGACCCCCTCAACTTCAGTGTGCTAAGCATTGTTGTCGAAGT
            IR29    (156)  CTCAAAGAAGACCCCCTCAACTTCAGTGTGCTAAGCATTGTTGTCGAAGT
              Ka    (156)  CTCAAAGAAGACCCCCTCAACTTCAGTGTGCTAAGCATTGTTGTCGAAGT
              KV    (156)  CTCAAAGAAGACCCCCTCAACTTCAGTGTGCTAAGCATTGTTGTCGAAGT
              NB    (156)  CTCAAAGAAGACCCCCTCAACTTCAGTGTGCTAAGCATTGTTGTCGAAGT
              Ni    (156)  CTCAAAGAAGACCCCCTCAACTTCAGTGTGCTAAGCATTGTTGTCGAAGT
             NSI    (156)  CTCAAAGAAGACCCCCTCAACTTCAGTGTGCTAAGCATTGTTGTCGAAGT
              Po    (156)  CTCAAAGAAGACCCCCTCAACTTCAGTGTGCTAAGCATTGTTGTCGAAGT
             SAL    (156)  CTCAAAGAAGACCCCCTCAACTTCAGTGTGCTAAGCATTGTTGTCGAAGT
    Ni-HKT1;4 Ex2   (166)  CTCAAAGAAGACCCCCTCAACTTCAGTGTGCTAAGCATTGTTGTCGAAGT
   Ni-HKT1;4 gDNA  (4501)  CTCAAAGAAGACCCCCTCAACTTCAGTGTGCTAAGCATTGTTGTCGAAGT

                              4551                                             4600
           FL478    (206)  TGTCAGGCAAGTCAGACTAAATGGTTTCTTATCTGAGAAAAAAAATGCAG
            IR29    (206)  TGTCAGGCAAGTCAGACTAAATGGTTTCTTATCTGAGAAAAAAAATGCAG
              Ka    (206)  TGTCAGGCAAGTCAGACTAAATGGTTTCTTATCTGAGAAAAAAAATGCAG
              KV    (206)  TGTCAGGCAAGTCAGACTAAATGGTTTCTTATCTGAGAAAAAAAATGCAG
              NB    (206)  TGTCAGGCAAGTCAGACTAAATGGTTTCTTATCTGAGAAAAAAAATGCAG
              Ni    (206)  TGTCAGGCAAGTCAGACTAAATGGTTTCTTACCTGAGAAAAAAAATGCAG
             NSI    (206)  TGTCAGGCAAGTCAGACTAAATGGTTTCTTATCTGAGAAAAAAAATGCAG
              Po    (206)  TGTCAGGCAAGTCAGACTAAATGGTTTCTTATCTGAGAAAAAAAATGCAG
             SAL    (206)  TGTCAGGCAAGTCAGACTAAATGGTTTCTTATCTGAGAAAAAAAATGCAG
    Ni-HKT1;4 Ex2   (216)  TGTCAG--------------------------------------------
   Ni-HKT1;4 gDNA  (4551)  TGTCAGGCAAGTCAGACTAAATGGTTTCTTACCTGAGAAAAAAAATGCAG

                              4601                                             4650
           FL478    (256)  ACCAAGTAAATTAACAATTTATCTGATGAAAACAGCAAAACACAAAGTAA
            IR29    (256)  ACCAAGTAAATTAACAATTTATCTGATGAAAACAGCAAAACACAAAGTAA
              Ka    (256)  ACCAAGTAAATTAACAATTTATCTGATGAAAACAGCAAAACACAAAGTAA
              KV    (256)  ACCAAGTAAATTAACAATTTATCTGATGAAAACAGCAAAACACAAAGTAA
              NB    (256)  ACCAAGTAAATTAACAATTTATCTGATGAAAACAGCAAAACACAAAGTAA
              Ni    (256)  ACCAAGTAAATTAACAATTTATCTGATGAAAACAGCAAAACACAAAGTAA
             NSI    (256)  ACCAAGTAAATTAACAATTTATCTGATGAAAACAGCAAAACACAAAGTAA
              Po    (256)  ACCAAGTAAATTAACAATTTATCTGATGAAAACAGCAAAACACAAAGTAA
             SAL    (256)  ACCAAGTAAATTAACAATTTATCTGATGAAAACAGCAAAACACAAAGTAA
    Ni-HKT1;4 Ex2   (222)  --------------------------------------------------
   Ni-HKT1;4 gDNA  (4601)  ACCAAGTAAATTAACAATTTATCTGATGAAAACAGCAAAACACAAAGTAA

                              4651                                             4700
           FL478    (306)  CACTTGTCAGGTATTTCTGAATCTTAATAATACACAATGAAAATAGTAAT
            IR29    (306)  CACTTGTCAGGTATTTCTGAATCTTAATAATACACAATGAAAATAGTAAT
              Ka    (306)  CACTTGTCAGGTATTTCTGAATCTTAATAATACACAATGAAAATAGTAAT
              KV    (306)  CACTTGTCAGGTATTTCTGAATCTTAATAATACACAATGAAAATAGTAAT
              NB    (306)  CACTTGTCAGGTATTTCTGAATCTTAATAATACACAATGAAAATAGTAAT
              Ni    (306)  CACTTGTCAGGTATTTCTGAATCTTAATAATACACAATGAAAATAGTAAT
             NSI    (306)  CACTTGTCAGGTATTTCTGAATCTTAATAATACACAATGAAAATAGTAAT
              Po    (306)  CACTTGTCAGGTATTTCTGAATCTTAATAATACACAATGAAAATAGTAAT
             SAL    (306)  CACTTGTCAGGTATTTCTGAATCTTAATAATACACAATGAAAATAGTAAT
    Ni-HKT1;4 Ex2   (222)  --------------------------------------------------
   Ni-HKT1;4 gDNA  (4651)  CACTTGTCAGGTATTTCTGAATCTTAATAATACACAATGAAAATAGTAAT

                              4701                                             4750
           FL478    (356)  GTTTTCTTTCGTGGAATCTGACGGATCTGCCCACATTTGCAGTGCATATG
            IR29    (356)  GTTTTCTTTCGTGGAATCTGACGGATCTGCCCACATTTGCAGTGCATATG
              Ka    (356)  GTTTTCTTTCGTGGAATCTGACGGATCTGCCCACATTTGCAGTGCATATG
              KV    (356)  GTTTTCTTTCGTGGAATCTGACGGATCTGCCCACATTTGCAGTGCATATG
              NB    (356)  GTTTTCTTTCGTGGAATCTGACGGATCTGCCCACATTTGCAGTGCATATG
              Ni    (356)  GTTTTCTTTCGTGGAATCTGACGGATCTGCCCACATTTGCAGTGCATATG
             NSI    (356)  GTTTTCTTTCGTGGAATCTGACGGATCTGCCCACATTTGCAGTGCATATG
              Po    (356)  GTTTTCTTTCGTGGAATCTGACGGATCTGCCCACATTTGCAGTGCATATG
             SAL    (356)  GTTTTCTTTCGTGGAATCTGACGGATCTGCCCACATTTGCAGTGCATATG
    Ni-HKT1;4 Ex3     (1)  ------------------------------------------TGCATATG
   Ni-HKT1;4 gDNA  (4701)  GTTTTCTTTCGTGGAATCTGACGGATCTGCCCACATTTGCAGTGCATATG
```

```
                        4751                                              4800
         FL478   (406)  GAAATGTGGGGTTCTCAATGGGCTACAGTTGCAGTAGACAGATCAATCCA
          IR29   (406)  GAAATGTGGGGTTCTCAATGGGCTACAGTTGCAGTAGACAGATCAATCCA
            Ka   (406)  GAAATGTGGGGTTCTCAATGGGCTACAGTTGCAGTAGACAGATCAATCCA
            KV   (406)  GAAATGTGGGGTTCTCAATGGGCTACAGTTGCAGTAGACAGATCAATCCA
            NB   (406)  GAAATGTGGGGTTCTCAATGGGCTACAGTTGCAGTAGACAGATCAATCCA
            Ni   (406)  GAAATGTGGGGTTCTCAATGGGCTACAGTTGCAGTAGACAGATCAATCCA
           NSI   (406)  GAAATGTGGGGTTCTCAATGGGCTACAGTTGCAGTAGACAGATCAATCCA
            Po   (406)  GAAATGTGGGGTTCTCAATGGGCTACAGTTGCAGTAGACAGATCAATCCA
           SAL   (406)  GAAATGTGGGGTTCTCAATGGGCTACAGTTGCAGTAGACAGATCAATCCA
  Ni-HKT1;4 Ex3    (9)  GAAATGTGGGGTTCTCAATGGGCTACAGTTGCAGTAGACAGATCAATCCA
 Ni-HKT1;4 gDNA (4751)  GAAATGTGGGGTTCTCAATGGGCTACAGTTGCAGTAGACAGATCAATCCA

                        4801                                              4850
         FL478   (456)  GACCATCTCTGC--------------------------------------
          IR29   (456)  GACCATCTCTGC--------------------------------------
            Ka   (456)  GACCATCTCTGC--------------------------------------
            KV   (456)  GACCATCTCTGC--------------------------------------
            NB   (456)  GACCATCTCTGC--------------------------------------
            Ni   (456)  GACCATCTCTGC--------------------------------------
           NSI   (456)  GACCATCTCTGC--------------------------------------
            Po   (456)  GACCATCTCTGC--------------------------------------
           SAL   (456)  GACCATCTCTGC--------------------------------------
  Ni-HKT1;4 Ex3   (59)  GACCATCTCTGCACAGATAAATGGACTGGTTTCGTAGGGAGATGGAGCGA
 Ni-HKT1;4 gDNA (4801)  GACCATCTCTGCACAGATAAATGGACTGGTTTCGTAGGGAGATGGAGCGA
```

Figure S4. Sequence comparison of *OsHKT1;4* genomic DNA fragments across several rice lines. Two regions of the *OsHKT1;4* gene were sequenced from genomic DNA in nine rice lines. The first region (~300 bp) covers the 3'-end of the first exon up until the 5'-end of the first intron and the second region (~500 bp) from the start of the second exon until the 5'-end of the third exon. The sequences obtained were aligned with the Ni-*OsHKT1;4* locus genomic sequence mined from public databases. No differences were observed in any of the lines analyzed besides a point mutation in the second intron of Nipponbare which may represent a *japonica* v. *indica* single nucleotide polymorphism. Sequence alignment was performed using Align X (Invitrogen). Homologous sequences are highlighted in yellow or blue. The conserved codon encoding a Val residue in position 344 of the OsHKT1;4 protein is highlighted in green. The abbreviations Po = Pokkali; NB = Nona Bokra; Ni = Nipponbare; KV = Kallurundai Vellai; Ka = Kalurundai; NSI = NSICRC106; and SAL = SAL208 stand for individual varieties, whereas IR29 and FL478 are full names of rice varieties.
