# Supplementary material for: A Two-Staged Model of Na+ Exclusion in Rice Explained by 3D Modeling of HKT Transporters and Alternative Splicing
Source: PLoS One. 2012 Jul 11;7(7):e39865. doi: 10.1371/journal.pone.0039865 (PMC3394774; doi:10.1371/journal.pone.0039865)
Supplement: Figure S5 — Sequences of OsHKT1;4 splice variants. A predicted 522 bp fragment spanning over the OsHKT1;4 transcript three exons was amplified by RT-PCR from RNA purified from salt treated Nipponbare and Pokkali sheath tissue. Three amplification products were recovered from each line: the predicted 522 bp fragment (HKT1;4) and two other fragments of superior sizes (HKT1;4-SV1 and HKT1;4-SV2). No sequence variation was observed between the fragments of similar size across the two lines (data not shown). Here, an alignment of the three splice forms is presented against the genomic sequence of the OsHKT1;4 gene (Genomic). The increased length of both splice variants is due to alternative splicing of the second intron. Whereas SV1 is splicing a second shorter intron, only one intron, the first, is spliced in SV2. Both variants are translated into an identical truncated protein due to the presence of an in frame STOP codon in the 5′-end of OsHKT1;4 second intron. Sequence alignment was performed using Align X (Invitrogen). Homologous sequences are highlighted in yellow or blue. The full length second intron is highlighted in blue. The conserved codon encoding a Val residue in position 344 of the OsHKT1;4 protein is highlighted in green. The in frame STOP codon in the second intron is highlighted in purple. The RT-PCR primers are highlighted in grey. The sequences of the primers used to perform the HPLC molecular count ratio between the three splice forms are underlined in black. (PDF) [file pone.0039865.s005.pdf]

```
                   1                                                50
   Genomic     (1) ATGCCGGCGCGGCGGTGCTGGATGCTGGCGGCGACGGTGGCGGCGTTCGT
HKT1;4-SV2     (1) ATGCCGGCGCGGCGGTGCTGGATGCTGGCGGCGACGGTGGCGGCGTTCGT
HKT1;4-SV1     (1) ATGCCGGCGCGGCGGTGCTGGATGCTGGCGGCGACGGTGGCGGCGTTCGT
    HKT1;4     (1) ATGCCGGCGCGGCGGTGCTGGATGCTGGCGGCGACGGTGGCGGCGTTCGT

                   51                                              100
   Genomic    (51) CGCCGTGCTGATGGCGCTGGTGTGCGGCATGGAGTGGGGCGGGGCGCTGC
HKT1;4-SV2    (51) CGCCGTGCTGATGGCGCTGGTGTGCGGCATGGAGTGGGGCGGGGCGCTGC
HKT1;4-SV1    (51) CGCCGTGCTGATGGCGCTGGTGTGCGGCATGGAGTGGGGCGGGGCGCTGC
    HKT1;4    (51) CGCCGTGCTGATGGCGCTGGTGTGCGGCATGGAGTGGGGCGGGGCGCTGC

                   101                                             150
   Genomic   (101) AGGGGATGAGCCCGTGGGAGAAGGTGGTGAACGCGCTGTTCCTCGCCGTG
HKT1;4-SV2   (101) AGGGGATGAGCCCGTGGGAGAAGGTGGTGAACGCGCTGTTCCTCGCCGTG
HKT1;4-SV1   (101) AGGGGATGAGCCCGTGGGAGAAGGTGGTGAACGCGCTGTTCCTCGCCGTG
    HKT1;4   (101) AGGGGATGAGCCCGTGGGAGAAGGTGGTGAACGCGCTGTTCCTCGCCGTG

                   151                                             200
   Genomic   (151) AACGCCCGGCACACCGGCGAGTCCACCGTCGACCTCTCCATCCTCGCGCC
HKT1;4-SV2   (151) AACGCCCGGCACACCGGCGAGTCCACCGTCGACCTCTCCATCCTCGCGCC
HKT1;4-SV1   (151) AACGCCCGGCACACCGGCGAGTCCACCGTCGACCTCTCCATCCTCGCGCC
    HKT1;4   (151) AACGCCCGGCACACCGGCGAGTCCACCGTCGACCTCTCCATCCTCGCGCC

                   201                                             250
   Genomic   (201) GGCCATCCTCGTGCTCTTCGTCCTCATGATGTGAGTGCTCTCTGTTTCTC
HKT1;4-SV2   (201) GGCCATCCTCGTGCTCTTCGTCCTCATGAT--------------------
HKT1;4-SV1   (201) GGCCATCCTCGTGCTCTTCGTCCTCATGAT--------------------
    HKT1;4   (201) GGCCATCCTCGTGCTCTTCGTCCTCATGAT--------------------

                   2901                                           2950
   Genomic  (2901) TCATATTCTGCTTCATCTTGCCTCCAGGTATCTACCTCCGTACACGACGT
HKT1;4-SV2   (231) ---------------------------GTATCTACCTCCGTACACGACGT
HKT1;4-SV1   (231) ---------------------------GTATCTACCTCCGTACACGACGT
    HKT1;4   (231) ---------------------------GTATCTACCTCCGTACACGACGT

                   2951                                           3000
   Genomic  (2951) GGTTCCCATTTGAAGAGAATTCCACTACTAAGGATAGTAATGCAGAGAAC
HKT1;4-SV2   (254) GGTTCCCATTTGAAGAGAATTCCACTACTAAGGATAGTAATGCAGAGAAC
HKT1;4-SV1   (254) GGTTCCCATTTGAAGAGAATTCCACTACTAAGGATAGTAATGCAGAGAAC
    HKT1;4   (254) GGTTCCCATTTGAAGAGAATTCCACTACTAAGGATAGTAATGCAGAGAAC

                   3001                                           3050
   Genomic  (3001) CAGGGAATCAGACTGCTCGAGAGTACACTTTTGTCACAACTCTCCTACCT
HKT1;4-SV2   (304) CAGGGAATCAGACTGCTCGAGAGTACACTTTTGTCACAACTCTCCTACCT
HKT1;4-SV1   (304) CAGGGAATCAGACTGCTCGAGAGTACACTTTTGTCACAACTCTCCTACCT
    HKT1;4   (304) CAGGGAATCAGACTGCTCGAGAGTACACTTTTGTCACAACTCTCCTACCT

                   3051                                           3100
   Genomic  (3051) GACCATCTTTGTCATTGCCATCTGCATCACCGAGAGAAGAAAGCTCAAAG
HKT1;4-SV2   (354) GACCATCTTTGTCATTGCCATCTGCATCACCGAGAGAAGAAAGCTCAAAG
HKT1;4-SV1   (354) GACCATCTTTGTCATTGCCATCTGCATCACCGAGAGAAGAAAGCTCAAAG
    HKT1;4   (354) GACCATCTTTGTCATTGCCATCTGCATCACCGAGAGAAGAAAGCTCAAAG

                   3101                                           3150
   Genomic  (3101) AAGACCCCCTCAACTTCAGTGTGCTAAGCATTGTTGTCGAAGTTGTCAGG
HKT1;4-SV2   (404) AAGACCCCCTCAACTTCAGTGTGCTAAGCATTGTTGTCGAAGTTGTCAGG
HKT1;4-SV1   (404) AAGACCCCCTCAACTTCAGTGTGCTAAGCATTGTTGTCGAAGTTGTCAGG
    HKT1;4   (404) AAGACCCCCTCAACTTCAGTGTGCTAAGCATTGTTGTCGAAGTTGTCAG-

                   3151                                           3200
   Genomic  (3151) CAAGTCAGACTAAATGGTTTCTTACCTGAGAAAAAAAATGCAGACCAAGT
HKT1;4-SV2   (454) CAAGTCAGACTAAATGGTTTCTTACCTGAGAAAAAAAATGCAGACCAAGT
HKT1;4-SV1   (454) CAAGTCAGACTAAATGGTTTCTTACCTGAGAAAAAAAATGCAGACCAAGT
    HKT1;4   (453) --------------------------------------------------
                   3201                                           3250
   Genomic  (3201) AAATTAACAATTTATCTGATGAAAACAGCAAAACACAAAGTAACACTTGT
HKT1;4-SV2   (504) AAATTAACAATTTATCTGATGAAAACAGCAAAACACAAAGTAACACTTGT
HKT1;4-SV1   (504) AAATTAACAATTTATCTGATGAAAACAGCAAAACACAAAGTAACACTTGT
    HKT1;4   (453) --------------------------------------------------
```

```
                   3251                                               3300
   Genomic  (3251) CAGGTATTTCTGAATCTTAATAATACACAATGAAAATAGTAATGTTTTCT
HKT1;4-SV2   (554) CAGGTATTTCTGAATCTTAATAATACACAATGAAAATAGTAATGTTTTCT
HKT1;4-SV1   (554) CAG-----------------------------------------------
    HKT1;4   (453) --------------------------------------------------
                   3301                                               3350
   Genomic  (3301) TTCGTGGAATCTGACGGATCTGCCCACATTTGCAGTGCATATGGAAATGT
HKT1;4-SV2   (604) TTCGTGGAATCTGACGGATCTGCCCACATTTGCAGTGCATATGGAAATGT
HKT1;4-SV1   (557) -----------------------------------TGCATATGGAAATGT
    HKT1;4   (453) -----------------------------------TGCATATGGAAATGT
                   3351                                               3400
   Genomic  (3351) GGGGTTCTCAATGGGCTACAGTTGCAGTAGACAGATCAATCCAGACCATC
HKT1;4-SV2   (654) GGGGTTCTCAATGGGCTACAGTTGCAGTAGACAGATCAATCCAGACCATC
HKT1;4-SV1   (572) GGGGTTCTCAATGGGCTACAGTTGCAGTAGACAGATCAATCCAGACCATC
    HKT1;4   (468) GGGGTTCTCAATGGGCTACAGTTGCAGTAGACAGATCAATCCAGACCATC
                   3401
   Genomic  (3401) TCTGC
HKT1;4-SV2   (704) TCTGC
HKT1;4-SV1   (622) TCTGC
    HKT1;4   (518) TCTGC
```

Figure S5. Sequences of *OsHKT1;4* splice variants. A predicted 522 bp fragment spanning over the *OsHKT1;4* transcript three exons was amplified by RT-PCR from RNA purified from salt treated Nipponbare and Pokkali sheath tissue. Three amplification products were recovered from each line: the predicted 522 bp fragment (HKT1;4) and two other fragments of superior sizes (HKT1;4-SV1 and HKT1;4-SV2). No sequence variation was observed between the fragments of similar size across the two lines (data not shown). Here, an alignment of the three splice forms is presented against the genomic sequence of the *OsHKT1;4* gene (Genomic). The increased length of both splice variants is due to alternative splicing of the second intron. Whereas *SV1* is splicing a second shorter intron, only one intron, the first, is spliced in *SV2*. Both variants are translated into an identical truncated protein due to the presence of an in frame STOP codon in the 5'-end of *OsHKT1;4* second intron. Sequence alignment was performed using Align X (Invitrogen). Homologous sequences are highlighted in yellow or blue. The full length second intron is highlighted in blue. The conserved codon encoding a Val residue in position 344 of the OsHKT1;4 protein is highlighted in green. The in frame STOP codon in the second intron is highlighted in purple. The RT-PCR primers are highlighted in grey. The sequences of the primers used to perform the HPLC molecular count ratio between the three splice forms are underlined in black.
