# Supplementary material for: A Two-Staged Model of Na+ Exclusion in Rice Explained by 3D Modeling of HKT Transporters and Alternative Splicing
Source: PLoS One. 2012 Jul 11;7(7):e39865. doi: 10.1371/journal.pone.0039865 (PMC3394774; doi:10.1371/journal.pone.0039865)
Supplement: Figure S6 — OsHKT1;4 protein sequence comparison. The Pokkali (Po) OsHKT1;4 transcript encoding a functional protein was fully sequenced and its nucleotide sequence translated into an amino acid sequence. Po-OsHKT1;4 encodes for a 500 amino acid protein which share 99.8% homology with the OsHKT1;4 protein from Nipponbare (Ni). The sole difference between the two functional OsHKT1;4 proteins is a R to Q substitution in position 110 in Pokkali. The structural modeling of the OsHKT1;4 protein predicts that this substitution is localized in the cytoplasm-exposed loop inter-connecting membrane -helices between the first two domains of the protein and thus does not affect Na+ transport rate. Ni-OsHKT1;4 and Po-OsHKT1;4 were aligned against a predicted truncated protein generated by the splice variants. This predicted splice variant protein is 47 amino acid residues shorter and differs from the full-length OsHKT1;4 protein in its 19 C-terminal residues. The protein sequences were aligned using Align X (Invitrogen). Homologous regions are highlighted in yellow or blue. The conserved Val residue in position 344 is highlighted in green. (PDF) [file pone.0039865.s006.pdf]

```
                                  1                                                 50
            Ni-HKT1;4      (1)   MPTSRRALAGGALSMHVAYFLAISCLGYGLLGVLKVREPGAAPRRIDRFF
            Po-HKT1;4      (1)   MPTSRRALAGGALSMHVAYFLAISCLGYGLLGVLKVREPGAAPRRIDRFF
  Ni-HKT1;4 truncated      (1)   MPTSRRALAGGALSMHVAYFLAISCLGYGLLGVLKVREPGAAPRRIDRFF
                                  51                                               100
            Ni-HKT1;4     (51)   TAVSAATVSSMSTVEMEVFSNGQLVVLTVLMLLGGEVFVSLVGLASKWSK
            Po-HKT1;4     (51)   TAVSAATVSSMSTVEMEVFSNGQLVVLTVLMLLGGEVFVSLVGLASKWSK
  Ni-HKT1;4 truncated     (51)   TAVSAATVSSMSTVEMEVFSNGQLVVLTVLMLLGGEVFVSLVGLASKWSK
                                  101                                              150
            Ni-HKT1;4    (101)   LRSDAMDRSRRVESHGDVALADIDGGDVENPTSSGEEAASRRRPMDADTL
            Po-HKT1;4    (101)   LRSDAMDRSQRVESHGDVALADIDGGDVENPTSSGEEAASRRRPMDADTL
  Ni-HKT1;4 truncated    (101)   LRSDAMDRSRRVESHGDVALADIDGGDVENPTSSGEEAASRRRPMDADTL
                                  151                                              200
            Ni-HKT1;4    (151)   RHNAVRALFYIVLAIFAVVHVVGAVAVAAYVLASPGARRTLGDKSLNTWT
            Po-HKT1;4    (151)   RHNAVRALFYIVLAIFAVVHVVGAVAVAAYVLASPGARRTLGDKSLNTWT
  Ni-HKT1;4 truncated    (151)   RHNAVRALFYIVLAIFAVVHVVGAVAVAAYVLASPGARRTLGDKSLNTWT
                                  201                                              250
            Ni-HKT1;4    (201)   FAVFTTVSTFSNCGFMPTNENMVVFKRDAPLQLLLVPQVLAGNTLFAPLL
            Po-HKT1;4    (201)   FAVFTTVSTFSNCGFMPTNENMVVFKRDAPLQLLLVPQVLAGNTLFAPLL
  Ni-HKT1;4 truncated    (201)   FAVFTTVSTFSNCGFMPTNENMVVFKRDAPLQLLLVPQVLAGNTLFAPLL
                                  251                                              300
            Ni-HKT1;4    (251)   AACVWAAAAATRREELVEMAREGGRAAAAGYAHLMPARRCWMLAATVAAF
            Po-HKT1;4    (251)   AACVWAAAAATRREELVEMAREGGRAAAAGYAHLMPARRCWMLAATVAAF
  Ni-HKT1;4 truncated    (251)   AACVWAAAAATRREELVEMAREGGRAAAAGYAHLMPARRCWMLAATVAAF
                                  301                                              350
            Ni-HKT1;4    (301)   VAVLMALVCGMEWGGALQGMSPWEKVVNALFLAVNARHTGESTVDLSILA
            Po-HKT1;4    (301)   VAVLMALVCGMEWGGALQGMSPWEKVVNALFLAVNARHTGESTVDLSILA
  Ni-HKT1;4 truncated    (301)   VAVLMALVCGMEWGGALQGMSPWEKVVNALFLAVNARHTGESTVDLSILA
                                  351                                              400
            Ni-HKT1;4    (351)   PAILVLFVLMMYLPPYTTWFPFEENSTTKDSNAENQGIRLLESTLLSQLS
            Po-HKT1;4    (351)   PAILVLFVLMMYLPPYTTWFPFEENSTTKDSNAENQGIRLLESTLLSQLS
  Ni-HKT1;4 truncated    (351)   PAILVLFVLMMYLPPYTTWFPFEENSTTKDSNAENQGIRLLESTLLSQLS
                                  401                                              450
            Ni-HKT1;4    (401)   YLTIFVIAICITERRKLKEDPLNFSVLSIVVEVVSAYGNVGFSMGYSCSR
            Po-HKT1;4    (401)   YLTIFVIAICITERRKLKEDPLNFSVLSIVVEVVSAYGNVGFSMGYSCSR
  Ni-HKT1;4 truncated    (401)   YLTIFVIAICITERRKLKEDPLNFSVLSIVVEVVRQVRLNGFLPEKKNAD
                                  451                                              500
            Ni-HKT1;4    (451)   QINPDHLCTDKWTGFVGRWSDSGKLILIFVMFFGRLKKFSMKGGKAWKLS
            Po-HKT1;4    (451)   QINPDHLCTDKWTGFVGRWSDSGKLILIFVMFFGRLKKFSMKGGKAWKLS
  Ni-HKT1;4 truncated    (451)   QVN-----------------------------------------------
```

Figure S6. OsHKT1;4 protein sequence comparison. The Pokkali (Po) *OsHKT1;4* transcript encoding a functional protein was fully sequenced and its nucleotide sequence translated into an amino acid sequence. Po-*OsHKT1;4* encodes for a 500 amino acid protein which share 99.8% homology with the OsHKT1;4 protein from Nipponbare (Ni). The sole difference between the two functional OsHKT1;4 proteins is a R to Q substitution in position 110 in Pokkali. The structural modeling of the OsHKT1;4 protein predicts that this substitution is localized in the cytoplasm-exposed loop inter-connecting membrane α-helices between the first two domains of the protein and thus does not affect Na^+ transport rate. Ni-OsHKT1;4 and Po-OsHKT1;4 were aligned against a predicted truncated protein generated by the splice variants. This predicted splice variant protein is 47 amino acid residues shorter and differs from the full-length OsHKT1;4 protein in its 19 C-terminal residues. The protein sequences were aligned using Align X (Invitrogen). Homologous regions are highlighted in yellow or blue. The conserved Val residue in position 344 is highlighted in green.
