# Supplementary material for: A Two-Staged Model of Na+ Exclusion in Rice Explained by 3D Modeling of HKT Transporters and Alternative Splicing
Source: PLoS One. 2012 Jul 11;7(7):e39865. doi: 10.1371/journal.pone.0039865 (PMC3394774; doi:10.1371/journal.pone.0039865)
Supplement: Figure S7 — Correlation between OsHKT1;4 sheath expression level and Na+ tissue concentration. (A) Correlation between sheath OsHKT1;4 expression level measured by qRT-PCR (3 biological reps per line; 3 technical reps per biological sample) and sheath Na+ concentration measured by flame photometry (n = 5) in 8 rice lines salt treated as per described in the methods section. (B) Similar correlation with blade Na+ concentration. No correlation were observed. The abbreviations NB = Nona Bokra; Ni = Nipponbare; KV = Kallurundai Vellai; Ka = Kalurundai; NSI = NSICRC106; and SAL = SAL208 stand for individual varieties, whereas IR29 and FL478 are full names of rice varieties. (PDF) [file pone.0039865.s007.pdf]

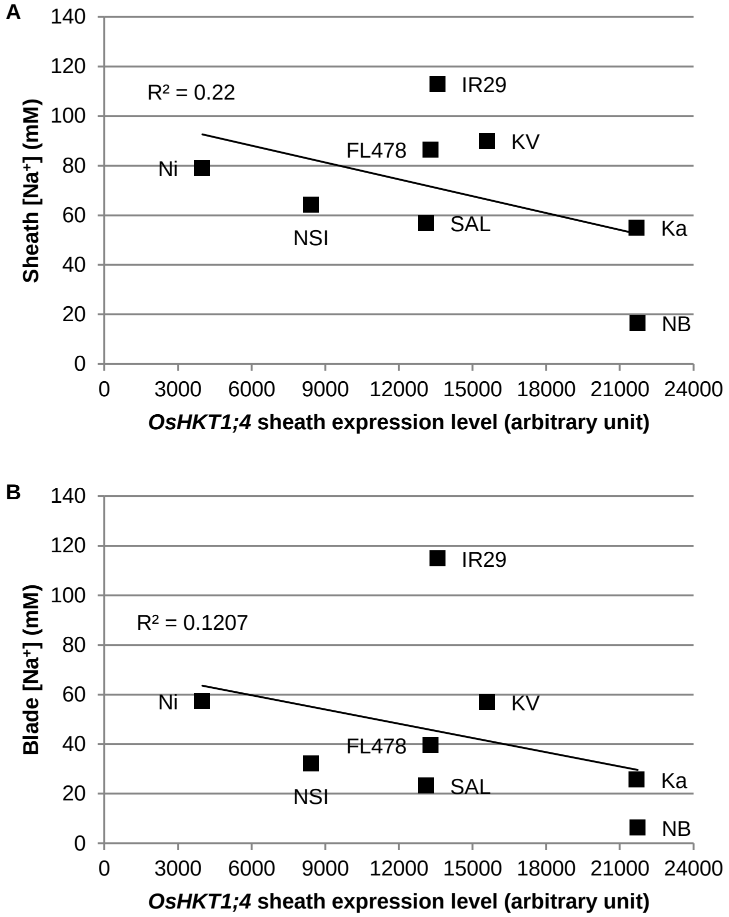

Figure S7. Correlation between *OsHKT1;4* sheath expression level and Na^+ tissue concentration. (**A**) Correlation between sheath *OsHKT1;4* expression level measured by qRT-PCR (3 biological reps per line; 3 technical reps per biological sample) and sheath Na^+ concentration measured by flame photometry (n = 5) in 8 rice lines salt treated as per described in the methods section. (**B**) Similar correlation with blade Na^+ concentration. No correlation were observed. The abbreviations NB = Nona Bokra; Ni = Nipponbare; KV = Kallurundai Vellai; Ka = Kalurundai; NSI = NSICRC106; and SAL = SAL208 stand for individual varieties, whereas IR29 and FL478 are full names of rice varieties.
